# Positive AMPA and Kainate Receptor Modulators and Their Therapeutic Potential in CNS Diseases: A Comprehensive Review

**DOI:** 10.3390/ijms26136450

**Published:** 2025-07-04

**Authors:** Alina Vialko, Paulina Chałupnik, Ewa Szymańska

**Affiliations:** 1 Department of Technology and Biotechnology of Drugs, Jagiellonian University Medical College in Kraków, PL 30-688 Kraków, Poland; alina.vialko@doctoral.uj.edu.pl (A.V.); chalupnik.paulina@gmail.com (P.C.); 2 Doctoral School of Medical and Health Sciences, Jagiellonian University Medical College, PL 31-530 Krakow, Poland

**Keywords:** AMPA receptors, kainate receptors, positive allosteric modulators, cognitive enhancers, preclinical studies, clinical trials

## Abstract

Ionotropic glutamate receptors—including *N*-methyl-d-aspartate (NMDA), α-amino-3-hydroxy-5-methyl-4-isoxazolepropionic acid (AMPA), and kainate receptors—play a pivotal role in excitatory signaling in the central nervous system (CNS), which is particularly important for learning and memory processes. Among them, AMPA and kainate receptors (known as ‘non-NMDA’ receptors) have gained increasing attention as therapeutic targets for various CNS disorders. Positive allosteric modulators (PAMs) of these receptors enhance their activity without directly activating them, offering a promising strategy to fine-tune glutamatergic signaling with potentially fewer side effects compared to orthosteric agonists. This review presents a comprehensive overview of recent advances in the development of AMPA and kainate receptor PAMs. We classify the most relevant modulators into main chemotype groups and discuss their binding modes, structure–activity relationships, and efficacy as determined through in vitro and in vivo studies. Additionally, we provide an overview of AMPA receptor PAMs that have entered into clinical trials over the past few decades. The increasing interest in kainate receptor PAMs is also mentioned, underlining their emerging role in future neuropharmacological strategies.

## 1. Introduction

Excitatory neurotransmission plays a fundamental role in communication between neurons in the central nervous system (CNS), driving key processes such as cognition, learning, and memory. This excitatory signaling is primarily mediated by the excitatory neurotransmitter l-glutamate, which activates several types of ionotropic (iGluRs) and metabotropic (mGluRs) receptors. The family of glutamate receptor cation channels is essential for most of the fast excitatory neurotransmission processes in the brain, and can be further divided into four main groups: *N*-methyl-d-aspartate receptors (NMDARs), α-amino-3-hydroxy-5-methyl-4-isoxazolepropionic acid receptors (AMPARs), kainate receptors (KARs), and orphan delta receptors. Among these subfamilies, AMPARs are responsible for mediating the rapid rise and decay of excitatory postsynaptic currents (EPSCs) and play a crucial role in both long-term potentiation (LTP) and long-term depression (LTD), two forms of synaptic plasticity that are essential for learning and memory.

AMPA receptors are widely distributed throughout the CNS, presenting high expression in the hippocampus and cortex, where they support cognitive functions, including learning and memory. In addition to their central role in synaptic transmission, AMPARs are found in glial cells—including astrocytes, oligodendrocyte precursor cells, and Bergmann glia—where they contribute to neuron–glia interactions and overall CNS functionality. They are also expressed in peripheral neurons, including those in the enteric nervous system of the colon [1]. Their prevalence within the CNS and their pivotal functions in fast excitatory neurotransmission and synaptic plasticity underscore the significance of AMPA receptors as a desirable target in the pursuit of novel therapeutic agents for neurodevelopmental, neuropsychiatric, and neurological conditions.

Kainate receptors are expressed in areas such as the hippocampus, amygdala, striatum, and spinal cord. Compared with AMPA and NMDA receptors, KARs are less understood and studied, in part due to a lack of specific pharmacological tools. In contrast to the other two subfamilies—which are predominantly located in postsynaptic compartments—KARs are distributed in both post- and presynaptic regions. In addition, these receptors have the capacity to function as both ionotropic and metabotropic receptors: they mediate excitatory postsynaptic currents while also bidirectionally modulating the release of both GABA and glutamate [2,3]. The latest findings regarding the biology of KA receptors indicate that they are involved in neurophysiological activity and play important roles in both health and disease, including conditions such as anxiety, schizophrenia, epilepsy, neuropathic pain, and migraine [3].

The regulation of AMPA and kainate receptor activities—particularly through the selective enhancement in receptor activation via allosteric modulation—is a highly desirable direction in the search for new drugs that act on the CNS. The development of positive allosteric modulators (PAMs) of AMPARs over the past three decades has contributed significantly to the understanding of the complex regulation of these receptors with respect to their subunit composition, RNA editing, alternative splicing, and auxiliary subunit interactions. Unlike most competitive agonists, PAMs have been shown to exhibit selectivity for specific AMPAR subpopulations. This characteristic renders them a promising class of therapeutic agents with a wide range of potential applications, including the treatment of cognitive and memory impairment, depression, and neurodegenerative diseases [1]. However, special attention must be paid to the possible proconvulsive effects of such compounds, which—as in the case of agonists—have been observed for some potent AMPAR potentiators. The present work presents a review of extensive in vitro and in vivo studies on PAMs of AMPA and kainate receptors with different structural scaffolds belonging to different chemical classes, including their therapeutic potential.

## 2. Structure and Pharmacological Modulation of AMPA and Kainate Receptors

AMPA and kainate receptors present modular structures consisting of several distinct domains that contribute to their assembly, function, and regulation. The large extracellular fragment of the receptor consists of two domains: the N-terminal domain (ATD), which is responsible for receptor assembly, trafficking, and allosteric regulation, and the ligand-binding domain (LBD)—also known as the agonist-binding domain—which binds glutamate and undergoes conformational changes leading to opening of the transmembrane ion channel. The transmembrane domain (TMD) of each subunit of the receptor is composed of four transmembrane helices (M1–M4) that form the channel pore, through which cations such as sodium (Na^+^) and potassium (K^+^) flow during receptor activation. The M2 segment forms a reentrant loop that contributes to the ion selectivity of the pore, including in some cases, calcium permeability. The TMD is critical for channel gating and conductance, and its structural arrangement is highly regulated by interactions with auxiliary proteins that modulate the gating kinetics of the receptor. The C-terminal domain (CTD), which is located intracellularly, regulates receptor localization, trafficking, and post-translational modifications such as phosphorylation, ubiquitination, and palmitoylation. This domain interacts with various intracellular proteins that regulate receptor cycling between the synaptic membrane and intracellular compartments—a process that is essential for synaptic plasticity and the maintenance of excitatory synapses. The CTD varies in length and sequence between different AMPA receptor subunits, contributing to functional diversity among the receptors [1].

AMPA receptors are tetrameric complexes comprising subunits from the set of GluA1, GluA2, GluA3, and GluA4, which are encoded by the *GRIA1*-*GRIA4* genes. Individual subunits can assemble into homo- or heterotetramers, with GluA2-containing heterotetramers being the most common in the CNS. The subunit composition of a receptor influences its functional properties, particularly its ion permeability and gating kinetics. The GluA2 subunit is especially significant in determining calcium permeability. RNA editing at the Q/R site of GluA2 changes the glutamine (Q) codon to arginine (R), rendering the receptor calcium-impermeable and resistant to polyamine blockade. In contrast, AMPARs lacking GluA2 are calcium-permeable, with implications for neuronal signaling and excitotoxicity. In addition to Q/R site editing, other subunits undergo RNA editing at the R/G site in the ligand-binding domain, affecting receptor desensitization and recovery [4].

Alternative splicing further diversifies AMPA receptor functions through the generation of flip and flop isoforms of the receptor subunits. These isoforms—which differ in the short region of the ligand-binding domain (Ser/Asn site)—are developmentally regulated and have distinct kinetic properties. Flip isoforms are predominant during early development and exhibit slower desensitization, while flop isoforms (which emerge later) show faster desensitization kinetics. The functional differences between these isoforms also impact the receptor’s sensitivity to pharmacological agents, including positive allosteric modulators [1].

For the process of functional regulation of AMPA receptors, auxiliary proteins—including transmembrane AMPA receptor regulatory proteins (TARPs), cornichon homologs (CNIHs), and cysteine-knot AMPA receptor modulating proteins (CKAMPs)—are integral parts of the receptor. These proteins associate with the receptor complex and modulate its gating kinetics, trafficking, and synaptic localization [5,6]. For instance, TARPs such as γ-2 (stargazin) and γ-8 play crucial roles in enhancing the surface expression of AMPA receptors, modulating receptor gating, and altering pharmacological properties. TARPs slow desensitization and enhance recovery, significantly influencing the time course of synaptic responses mediated by AMPA receptors. In addition to TARPs, CNIHs and other auxiliary subunits contribute to the stabilization and regulation of AMPA receptor activity in specific neuronal circuits [5]. Auxiliary proteins are expressed in a region-specific manner, which provides a mechanism for fine-tuning AMPAR function in different brain regions, as exemplified by the TARPs: γ-2 is essential for AMPAR function in cerebellar granule cells, while γ-8 modulates receptor activity in hippocampal neurons. This region-specific regulation significantly impacts synaptic function, opening up new possibilities for therapeutic strategies in the treatment of neurological diseases [6,7].

Kainate receptors are tetramers composed of the subunits GluK1-GluK5, of which the ‘low affinity’ subunits GluK1-GluK3 can form homo- or heteromeric functional ion channels, while the ‘high affinity’ subunits GluK4 and GluK5 form functional channels only in heteromeric assembly with one of the GluK1-GluK3 [3,8]. The subunit composition of KARs varies across regions, with GluK1 predominantly expressed in interneurons, GluK2 in glutamatergic neurons, and GluK3 in a variety of neuronal types [3]. KARs participate in frequency-dependent modulation of synaptic transmission and contribute to excitatory and inhibitory signaling in regions such as the striatum and cerebellum. Like AMPARs, KARs undergo RNA editing, which affects their ion permeability and receptor properties, although the functional significance of these changes is still not fully understood [1].

**Figure 1:**
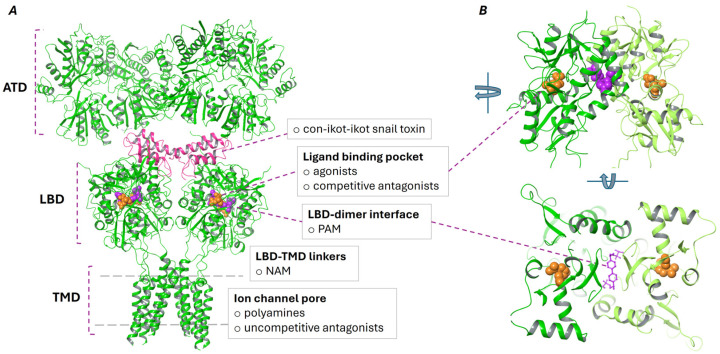
(**A**) X-ray structure (PDB ID: 4U5D) of the full-length GluA2 AMPA receptor (green cartoon) in complex with con-ikot-ikot snail toxin (pink cartoon), the partial agonist KA (orange spheres), and the positive allosteric modulator **49b** (magenta spheres). (**B**) Enlarged perpendicular views of the LBD–dimer interface (green cartoon) with KA (orange spheres) and one molecule of PAM **49b** (magenta spheres/thin sticks) bound at the dimer interface.

The resolution of numerous crystal structures of various AMPA and KA receptor subunits, conformational states, and full-length receptors has significantly advanced our understanding of the architecture, ligand binding, and modulation of these receptors. In particular, many studies have focused on the GluA2 AMPAR subunit, for which approximately 250 crystal structures of the LBD and full-length receptor are available in the Protein Data Bank (PDB). These data provide critical insights into interactions between the receptor and a wide range of ligands and modulators, enabling the identification of distinct binding sites (Figure 1). Additionally, heteromeric assemblies such as GluA2/GluA3 and GluA2/GluA4 have been characterized, revealing unique conformational features and subunit arrangements that contribute to the functional diversity of AMPA receptors [1].

Comparative analysis of LBD complexes with ligands bound at the orthosteric binding site has revealed that agonists typically induce conformational closure of the LBD, correlating with the efficacy of receptor activation. Conversely, competitive antagonists occupy the same site without triggering the conformational changes necessary for ion channel opening [9]. Negative allosteric modulators (NAMs) of AMPA and KA receptors—also known as non-competitive antagonists—diminish receptor activity by preventing the conformational changes that lead to ion channel opening upon agonist binding. It has been demonstrated that NAMs such as perampanel, GYKI 52466, GYKI 53655, and talampanel bind to a linker region between the LBD and TMD [1]. In contrast, uncompetitive competitive antagonists—including polyamine-based compounds—target the ion channel pore itself, thus blocking ion flow [10].

Positive allosteric modulators of AMPARs and kainate receptors are among the most intensively studied modulatory agents, due to their high therapeutic potential [11]. These compounds prolong receptor activation induced by an agonist by affecting the processes of desensitization and deactivation of the ion channel, thereby amplifying neurotransmitter effects at postsynaptic receptors [12]. Desensitization refers to the rapid closure of the ion channel and restriction of ion conductance that occurs as a result of prolonged stimulation with a bound agonist; meanwhile, during deactivation, the agonist leaves the binding site, and the receptor adopts a resting state [13,14]. In contrast to agonists, PAMs allow for more selective enhancement in synaptic functions, as their effects depend on the presence of endogenous ligands [1].

Structurally, PAMs bind at the LBD–dimer interface with one or two molecules (Figure 1), resulting in the stabilization of the agonist-bound receptor conformation, slowing down ion channel deactivation, and reducing the rate of desensitization [13,15,16,17,18]. According to Ptak et al., at least five overlapping binding subsites can be distinguished on the binding surface for PAMs: one central subsite and two pairs of symmetrical sites located on adjacent monomers [13,16].

While most PAMs exhibit selectivity for the flip isoform, exceptions such as PEPA and aniracetam prefer the flop isoform [1]. Furthermore, it has been demonstrated that certain compounds exert an indirect influence on AMPA receptor activity; for instance, minocycline modulates the functionality of AMPA receptors by increasing the phosphorylation of the GluA1 subunit [19]. Furthermore, distinctive regulatory mechanisms have been identified. Con-icot-icot—a conotoxin derived from *Conus striatus*—has been shown to act as a PAM by binding between the ATD and LBD layers, forming extensive contacts with all four LBD subunits and stabilizing the LBD gating ring, thereby preventing desensitization. This results in sustained activation of the receptor, leading to overexcitation and toxicity [18,20].

## 3. Positive Allosteric Modulators of AMPA and Kainate Receptors

### 3.1. Benzamides and Related Structures

**Figure 2:**
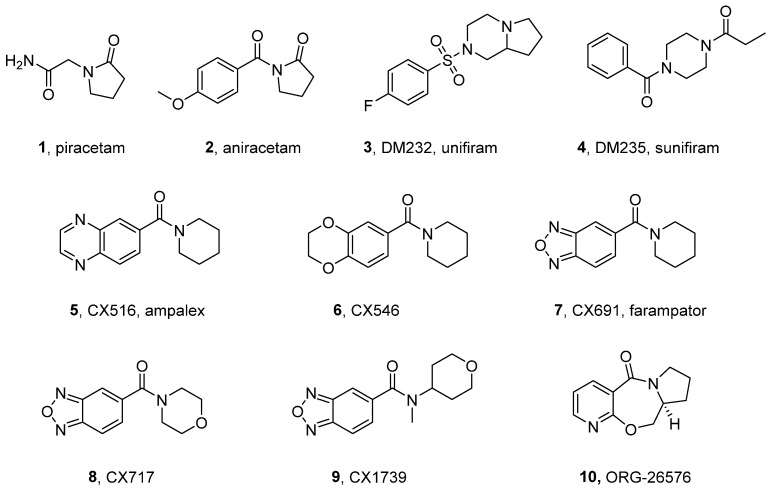
Benzamide-type AMPA receptor modulators.

Benzamide derivatives have emerged as a pharmacologically significant class of AMPAR modulators, with their origins traced to piracetam and aniracetam—some of the earliest nootropics (**1** and **2**, Figure 2) [21,22,23]. In vivo preclinical studies have indicated the potential efficacy of aniracetam in the treatment of mild to moderate cognitive and motor impairment in patients with stroke and Alzheimer’s disease (AD), alleviating symptoms associated with attention deficit hyperactivity disorder (ADHD), depression, sleep disturbances, autism spectrum disorder, and the negative symptoms of schizophrenia [23]. In clinical trials, **2** showed high potency when tested as monotherapy or in combination with cholinesterase inhibitors in patients with cognitive impairment [24]. Research on aniracetam has recently been reinitiated. In an experimental model of ischemic stroke, **2** reduced inflammation and increased brain-derived neurotrophic factor (BDNF) when combined with perampanel (a non-competitive AMPAR antagonist) [25]. It has been suggested that **2** may prevent the production and accumulation of amyloid-β plaques by increasing α-secretase activity through stimulation of mGluRs [26]. These recent findings—together with the favorable tolerability profile, safety, and few drug interactions—make aniracetam a good candidate for the prevention and treatment of Alzheimer’s disease. Additional studies have also demonstrated aniracetam’s potential efficacy in preventing and reversing the development of acute antinociceptive tolerance to morphine [27], as well as its positive effects on the cognitive deficits associated with Fetal Alcohol Syndrome (FASD) [28,29].

The structure of piracetam formed the basis for the development of unifiram (**3**), which possesses a condensed oxopyrrolidine with a piperazine ring in its structure, as well as its open-ring pyrrolidinone derivative sunifiram (**4**) [30,31,32]. Both compounds exhibit the ability to reverse NBQX-induced memory deficits, with unifiram also enhancing AMPA receptor-dependent currents in rat hippocampal CA1 slices (EC_50_ of approximately 27 nM). However, in the case of recombinant AMPA receptors (GluA1/GluA2), no enhancement in the AMPAR response was observed. Studies in animal models have shown that **3** and **4** at 0.001 mg/kg i.p. were effective in reversing scopolamine-induced memory impairment (passive avoidance test in mice), and at 0.1 mg/kg i.p., were effective in preventing scopolamine-induced amnesia in the Morris water maze in rats. However, when administered as monotherapy, both compounds were found to be inactive [30,31,32]. The exact mechanism of action of both compounds is not well understood, and despite their promising properties, they were not further developed in clinical trials. Despite the lack of clinical approval and little human data, both unifiram and sunifiram have appeared on the unregulated market as so-called ‘smart drugs,’ raising concerns about their safety, efficacy, and potential misuse [33].

Based on the structural formula of aniracetam, RespireRx Pharmaceuticals (formerly Cortex Pharmaceuticals) has developed a series of CX compounds (‘ampakines’), some of which have been clinically tested (for further details, see Section 4). The introduction of a heterocyclic ring fused to the benzamide core represented the initial structural modifications in this series, which eventually led to the development of the first-generation benzamide-type modulators CX516 (**5**, ampalex) and CX546 (**6**). The mechanism of action of CX516 involves enhancing synaptic responses through increasing the amplitude of EPSCs and prolonging their duration. This effect is achieved primarily through the selective acceleration of AMPA receptor channel opening, resulting in an amplified response while exerting minimal impact on receptor desensitization and deactivation kinetics [34]. Structural analysis of CX516 in complex with the ligand-binding domain of the GluA2 receptor (PDB ID: 4IY5) has suggested that **5**, like aniracetam, binds at the dimer interface center, near the region hinged between the L1 and L2 lobes [35]. In preclinical studies, the compound has demonstrated notable therapeutic efficacy, particularly in animal models of intellectual disability (5 mg/kg for 5 days) [36], as well as hyperactivity commonly associated with schizophrenia and autism spectrum disorders (10-40 mg/kg) [37]. CX516 administration at 5 mg/kg i.p. for 1 week alleviated chronic 20% (m/V) ethanol-induced anxiety- and depressive-like behaviors in mice [38]. Furthermore, chronic administration of CX516 (at 10 mg/kg i.p.) in a mouse model of sleep deprivation prevented the development of anhedonia. At the same time, CX516 showed a trend toward cognitive decline in the novel object recognition (NOR) test and anxiogenic effects in the elevated plus maze test. A proposed explanation for these findings suggests that changes in the postsynaptic AMPAR profile induced by chronic sleep deprivation may have prevented the anxiolytic effect produced by ampakines [39].

CX546 prolongs the duration of the synaptic response by slowing the deactivation of AMPA receptor currents (10-fold) and attenuating receptor desensitization. These actions result in an increased synaptic response amplitude and prolong depolarization in neurons [34]. Preclinical studies have shown that CX546 can enhance the efficacy of morphine in managing pain, suggesting a possible synergistic effect [40]. When administered independently, **6** exhibited analgesic properties in models of neuropathic and inflammatory pain [41]. Moreover, CX546 promoted the survival and proliferation of neural stem/progenitor cells (NSPCs) and reduced oxidative stress-induced cell death, indicating its utility in the field of neuro-regenerative stem cell therapies [42]. Further studies have confirmed the effectiveness of CX546 in reversing opioid- and barbiturate-induced respiratory depression without compromising their analgesic effects [42], and it has been found to prevent anesthesia-induced cognitive deficits [43].

Farampator (ORG-24448, CX691, **7**)—another molecule developed under the RespireRx license [44]—exhibited higher PAM potency in comparison with CX516 and CX546. Similarly to CX516 and in contrast to CX546, farampator only moderately offsets desensitization in hippocampal patches, indicating that it should be classified into the ‘low-impact’ category of ampakines, that is, compounds that have a reduced effect on synaptic currents. This feature, observed for certain ampakines, is thought to be related to the lack of seizure-inducing effects reported for ‘high-impact’ AMPAR-positive modulators such as cyclothiazide, which fully offset receptor desensitization and enhance agonist binding affinity [45]. In animal models, CX691 in small doses demonstrated antipsychotic activity, reduced amphetamine- and methamphetamine-induced locomotor activity, and showed synergistic effects with antipsychotics such as clozapine and olanzapine. When administered in supratherapeutic doses in rats, farampator did not result in the occurrence of cataleptic activity, as has been observed with haloperidol under analogous conditions [45]. In spatial learning and memory models based on rats, **7** demonstrated procognitive properties and enhanced BDNF protein levels in the hippocampal tissue of AD rats in comparison to untreated animals [46]. The compound’s ability to penetrate the blood–brain barrier, in conjunction with the findings of preclinical studies, led to its categorization as a promising clinical candidate for addressing the cognitive impairments associated with schizophrenia and Alzheimer’s disease (see Section 4).

The substitution of the piperidine ring with morpholine resulted in the discovery of CX717 (**8**) [44]. In vitro, **8** moderately attenuated AMPAR desensitization and increased the steady-state glutamate-induced peak current in hippocampal CA1 neurons to 25% of the peak amplitude, with an EC_50_ of 3.4 µM. In vivo studies demonstrated that **8** increased synaptic transmission in a dose-dependent manner and enhanced LTP induction at a dose of 2 mg/kg, increasing the field excitatory postsynaptic potential (EPSP) amplitude at the dentate bend by up to 21% in anesthetized rats. In behavioral models, CX717 reduced amphetamine-induced hyperlocomotion at doses of 1–3 mg/kg, demonstrating greater efficacy compared to CX516 [47]. Extensive preclinical evaluations have also indicated that **8**, similarly to other low-impact ampakines, can reverse opioid-induced respiratory depression, alleviate memory deficits associated with sleep deprivation, and improve respiratory function after spinal cord injury at doses of 5–30 mg/kg [47,48,49,50,51]. CX717 has been shown to enhance respiratory motor output when delivered intrathecally, producing a sustained increase in phrenic nerve activity without affecting cardiovascular function. Additionally, systemic CX717 administration prior to a single hypoxic episode significantly amplified phrenic motor facilitation (pMF)—a form of respiratory neuroplasticity typically requiring repeated hypoxia. These findings suggest that CX717 can potentiate respiratory neural drive and may be particularly effective when used in a timing-sensitive manner, offering therapeutic potential for hypoxia-based neurorehabilitation strategies [52,53]. Furthermore, the administration of 20 mg/kg CX717 i.p. was found to be effective in the forced swim test, demonstrating rapid but short-lasting antidepressant-like action presumably caused by increases in BDNF and p11 [54].

CX1739 (**9**), along with CX516, CX691, and CX717, belongs to the class of potent low-impact ampakines with only modest effects on AMPAR desensitization, a minimized risk of epileptogenic activity, and a broad therapeutic window. This compound has been found to be 40–100-fold more potent compared to CX516. In animal models, CX1739 enhanced long-term potentiation in rats in a dose-dependent manner and, at doses of 0.03–18 mg/kg, significantly improved memory and cognition in several tests, including the NOR test, the win shift radial arm maze, and the five-choice serial reaction time task [55]. In addition, **9** has been shown to rapidly reverse opioid-induced respiratory depression without reducing analgesia and attenuated amphetamine-induced locomotor hyperactivity [55,56]. In safety studies in rats, CX1739 showed no adverse effects up to 2000 mg/kg. These findings allowed both CX717 and its successor, CX1739, to be classified as good and safe clinical candidates for the treatment of dementia, ADHD, and other neuropsychiatric disorders or opiate-induced respiratory depression (see Section 4).

A novel analog of CX1739, LCX001—which contains a 4-hydroxycyclohexyl group in its structure instead of the tetrahydropyran found in CX1739—effectively prevented and rescued opioid-, propofol-, and pentobarbital-induced respiratory depression at 10 mg/kg intravenously. In contrast to other typical ampakines (which do not affect analgesia), LCX001 also exhibited antinociceptive effects in rodent models, making it a potential candidate for the treatment of pain and respiratory depression [57].

ORG-26576 (**10**), another example of benzamide-type AMPA modulators, was developed by RespireRx Pharmaceuticals and later licensed by Organon. Preclinical studies have highlighted its efficacy in enhancing the release of BDNF and producing nootropic effects [58,59]. In the behavioral Morris water maze model, ORG-26576 significantly improved both early initial learning in rats and spatial memory storage/retrieval at doses of 3 and 10 mg/kg (twice daily) [60]. The compound has entered into clinical trials to assess its potential to treat ADHD and major depressive disorder (see Section 4).

**Figure 3:**
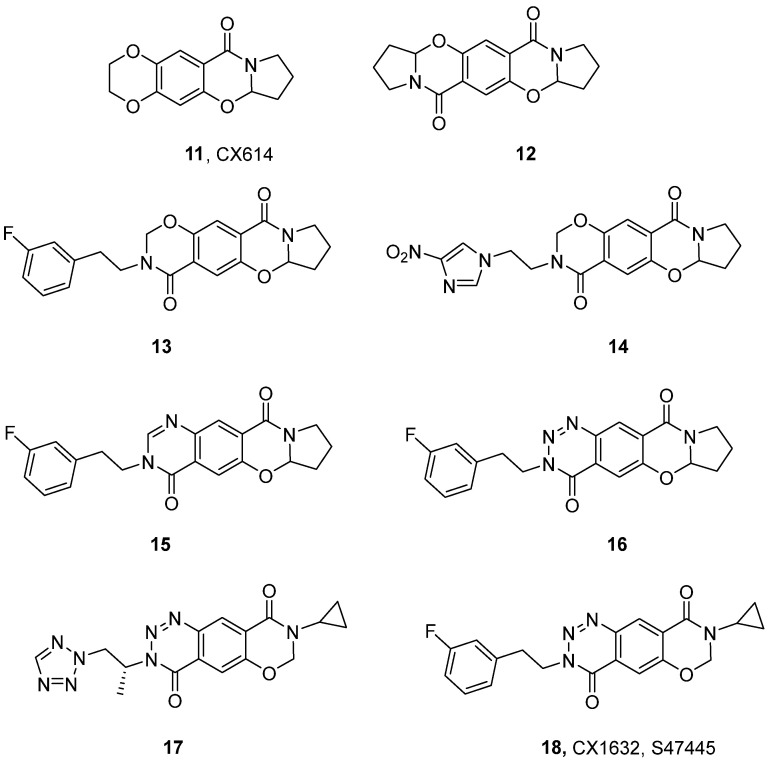
Benzamide-type AMPA receptor modulators derived from CX614.

The hydrolytic instability of aniracetam’s imide structure has been identified as the primary factor underlying its relatively brief in vivo duration of action. To address this, a conformationally rigid tetracyclic benzoxazinone analog (CX614, **11**, Figure 3) has been developed. This modification involved stabilizing the molecule by introducing a heteroatom-mediated additional ring, bridging the pyrrolidine and benzene rings to enhance both affinity and metabolic stability. The crystal structure of CX614 in complex with GluA2-LBD (PDB ID: 2AL4) exhibited a binding mode consistent with that previously observed for aniracetam and CX516, resulting in the stabilization of a closed, agonist-bound conformation of the ligand-binding core. However, due to its larger surface area and greater number of formed interactions, CX614 was found to bind more tightly than aniracetam [15]. Like aniracetam, CX614 has been reported to effectively inhibit receptor desensitization, slow deactivation, and show selectivity for the flop isoform [15,61]. Additionally, it enhanced the amplitude and duration of excitatory postsynaptic potentials in the hippocampus and elicited EPSCs in neuronal cultures. In preclinical studies, CX614 has demonstrated significant potential to induce BDNF [62] and potentiate the effects of antidepressants (i.e., imipramine and reboxetine) [63]. Furthermore, CX614 reduced the symptoms of irregular and elevated respiratory rate in animals with Parkinson’s disease and increased respiratory rate in healthy animals [64]. It also demonstrated oncolytic properties, reducing the viability of glioblastoma cells when administered concomitantly with fluoxetine [65].

Based on the structure of CX614, researchers have developed a series of ampakines and evaluated their ability to potentiate AMPAR-mediated currents using patch clamp electrophysiology in cultured rat embryonic hippocampal neurons [66]. The assessment focused on determining the EC_2x_ values, representing the concentration of each compound required to produce a two-fold increase in the AMPA receptor-mediated current amplitude (Table 1) [67]. The primary modification focused on the synthesis of bis-benzoxazinones, of which the ‘inverted’ analog **12** demonstrated significantly higher potency in vitro compared to CX614 [67]. Subsequent modifications included elimination of the pyrrolidine ring and the introduction of additional alkyl, arylalkyl, or heteroarylalkyl side chains at the nitrogen atom in the oxazinone ring. This resulted in analogs **13** and **14**, which exhibited substantially higher potency at AMPA receptors in comparison to **12** (Table 1) [68]. Furthermore, **13** was selected as a starting point for structural modifications consisting of substituting the oxazinone ring with either a pyrimidinone (**15**) or triazinone (**16**) ring. The resulting compounds exhibited enhanced AMPAR potentiation effects both in vitro and in vivo, as well as high metabolic stability and up to 100% oral bioavailability in rats [69].

Further studies have shown that the removal of the second pyrrolidine ring, the introduction of a short cyclopropyl chain, and an additional 2-substituted tetrazole at the side chain (**17**) are optimal for in vivo activity, significantly facilitating the induction of hippocampal LTP in rats at a dose of 1 mg/kg i.p. [69,71]. Consistently, the cyclopropyl derivative of **16**—CX1632 (**18**, S47445, tulrampator)—represents a potent and AMPAR-selective PAM, with slightly higher potency at the flop splice isoform of GluA1 receptor subunits when compared to other recombinant AMPA receptor subtypes [70]. In vivo, the administration of CX1632 (0.3 mg/kg) demonstrated procognitive effects in the novel object recognition and T-maze models, improving both episodic and spatial working memory in adult rodents. Additionally, the compound did not cause any significant changes in spontaneous locomotion or general behavior, and did not induce seizures even at doses of up to 1000 mg/kg [70]. In further preclinical studies, CX1632 has been shown to possess a significant ability to increase synaptic plasticity in older mice [72] and neurotrophin levels in both the hippocampus and prefrontal cortex of aged rats [73]. These encouraging findings, in conjunction with its broad therapeutic window, have enabled the compound to be classified as a potential treatment for Alzheimer’s disease and depression associated with dementia for the purpose of clinical trials (see Section 4).

### 3.2. Benzothiadiazines and Ring-Fused Thiadiazines

**Figure 4:**
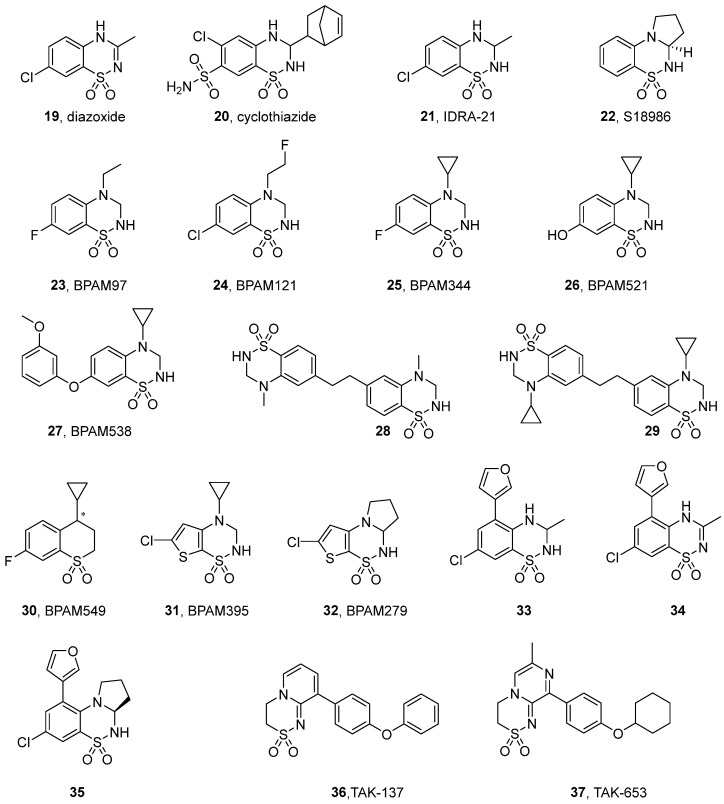
Benzothiadiazine-type AMPA receptor modulators.

Benzothiadiazine derivatives are among the most extensively studied classes of positive allosteric modulators of AMPA receptors. Early representatives of this category include diazoxide (**19**, Figure 4), cyclothiazide (CTZ, **20**), and IDRA-21 (**21**) [23]. In preclinical studies, the potent proconvulsant activity of CTZ has been demonstrated [74,75]. Interestingly, cyclothiazide has also been found to negatively modulate GABA_A_ and mGluR1 receptors [76,77]. IDRA-21 administration led to substantial enhancements in learning and memory functions [23]; however, it has been shown that the co-administration of the compound together with glutamate can lead to the death of cultured rat hippocampal neurons. In vivo, **21** has been observed to increase in vivo CA1 neuron loss produced via 10 min of global ischemia in rats, raising the risk of aggravation of ischemic neuronal damage [78].

Research into (hetero)aromatic thiadiazines—encompassing benzothiadiazines, pyridine thiadiazines, and thienothiadiazines—has been predominantly conducted and patented by Servier and the Laboratory of Medicinal Chemistry at the University of Liège. Comprehensive SAR analyses of these series of compounds have been exhaustively reviewed by Pirotte [11]. Among others, saturated thiadiazines demonstrated enhanced efficacy in comparison to their unsaturated counterparts, and replacement of the benzene ring with pyridine or thiophene significantly improved the pharmacological properties of the compounds. Within the described series of tricyclic thiadiazine dioxides, Servier’s compound S18986 (**22**)—a selective AMPA receptor modulator—showed significant potency in vitro (Table 2). In behavioral animal models, the compound was effective in preventing scopolamine-induced amnesia in a passive avoidance test and facilitated a form of episodic memory in rats in the object recognition test [79,80]. Lockhart et al. have shown that S18986 selectively acted on AMPA receptors and potently enhanced (*S*)-AMPA-induced release of noradrenaline in both rat hippocampal and frontal cortex slices, which can contribute to the cognition-enhancing actions of **22** [79]. The promising findings from preliminary studies led to the initiation of clinical trials focused on the treatment of mild cognitive impairment (MCI), a prodromal stage of Alzheimer’s disease (see Section 4).

Subsequent research has focused on direct derivatives of IDRA-21, with modifications such as the replacement of the methyl group at position 3 with short alkyl chains or the introduction of such chains into position 4 having resulted in a significant improvement in AMPA receptor activity (BPAM97, **23**) [81]. The incorporation of monofluoroethyl chains at position 4 (BPAM121, **24**) [82] further improved the pharmacokinetic properties, while cyclopropyl substitutions led to compounds such as BPAM344 (**25**) and BPAM521 (**26**), which exhibited higher potency and stability (Table 2 and Table 3) [83]. Structural and functional studies have provided detailed information on the interactions between these derivatives and individual AMPA and KA receptor subtypes. In contrast to the majority of developed PAMs, both BPAM344 and BPAM521 were shown to exert a strong positive modulatory effect on currents mediated by the homomeric KARs GluK1_b_, GluK2_a_, and GluK3_a_, significantly increasing the peak amplitudes of kainate receptor currents (in the range of 5- to 59-fold, depending on the subunit type; see Table 3) and markedly reducing the desensitization kinetics, with only a minor effect on the deactivation course. The varying magnitude of potentiation induced by BPAM344 across different homomeric KARs appears to correlate with their distinct desensitization properties. Specifically, the GluK3 receptor subtype, which is highly desensitizing, exhibited a 10-fold higher response to BPAM344 when compared to GluK1. X-ray crystallographic analyses of GluK1-GluK3 complexes (PDB ID: 5MFQ, 8R32, 8BSU) revealed a conserved BPAM344 binding mode at the dimer interface, stabilizing the receptor in an active-like conformation when co-bound with l-glutamate [86,87,88]. This mode of binding has been shown to significantly attenuate desensitization kinetics, thereby enhancing receptor activity [86]. Moreover, complementary cryo-electron microscopy studies have revealed that BPAM344 stabilizes GluK2 in a closed conformation, even in the absence of an agonist or in the presence of a competitive antagonist [89]. These findings highlight the potential of BPAM344 to modulate receptor states beyond traditional activation, suggesting its multifaceted role in KAR regulation [89].

More recently, a radiolabeled analog of BPAM344 ([^18^F]AMPA-2109) was successfully obtained as a positron emission tomography (PET) ligand, demonstrating good blood–brain barrier (BBB) permeability and high brain uptake in rodents and non-human primates. However, [^18^F]AMPA-2109 did not show proper specific binding, underscoring the need for further investigations in this field [90].

**Table 3:** Potentiation effects of benzothiadiazine-type modulators for selected recombinant homomeric iGluRs (in brackets: maximum potentiation effect observed at a specific modulator concentration).

Compound	EC_50_ [μM]			
	GluA2	GluK1	GluK2	GluK3
BPAM121 (**24**)	20.4 *^a^*	nd	no potentiation effect at 300 μM *^b^*	nd
BPAM344 (**25**)	0.81 *^a^* (5-fold at 100 μM) *^b^*	26 *^c^* (5-fold at 100 μM) *^b^*	79 *^b^* 75 *^c^* (15-fold at 100 μM) *^b^*	639 *^c^* (59-fold at 100 μM) *^b^*
BPAM521 (**26**)	2.5 *^d^*	nd	159 *^b^* (12-fold at 300 μM) *^b^*	nd
BPAM538 (**27**)	0.002 *^a^* (46% at 100 μM) *^e^*	58 *^e^* (130% at 100 μM) *^e^*	nd (32% at 100 μM) *^e^*	nd
**28**	0.0134 *^f^*	nd	nd	nd
**29**	0.0014 *^f^*	nd	nd	nd

*^a^* effects on the calcium flux induced by 1 mM glutamate in HEK293 cells expressing recombinant GluA2(*Q*)_o_ receptor, Ref. [91]; *^b^* effects on glutamate-evoked currents (1–10 mM) in HEK293 cells expressing GluK1_b_, GluK2(*Q*)_a_, GluK3_a_, or GluA1_i_ receptors, whole-cell recordings, Ref. [86]; *^c^* effects on KA-induced (100 μM) fluorescence in stable GT-HEK293 cell lines expressing GluK1(*Q*)_1b_, GluK2(*VCQ*)_2a_, and GluK3_a_ receptors in the presence of 100 μM kainate, Ref. [88]; *^d^* effects on (*S*)-glutamate-induced (10 µM) responses at homomeric rat GluA2(*Q*)_i_ expressed in *Xenopus laevis* oocytes, voltage clamp assay, Ref. [92]; *^e^* effects on the glutamate-induced (10 mM) current in HEK293 cell line expressing recombinant GluK1(*Q*)_b_, GluK2(*VCQ*)_a_, and GluA2(*Q*)_i_ receptor, electrophysiological recordings, Ref. [93]; *^f^* effects on the calcium flux induced by 1 mM glutamate in HEK293 cells expressing recombinant GluA2(*Q*)_o_ receptor, Ref. [94]; nd—not determined.

Goffin et al. introduced a mono-substituted 7-phenoxy group into the benzothiadiazine dioxide scaffold, resulting in an active analog, BPAM538 (**27**), that potentiates glutamate’s effects on AMPA receptors with nanomolar range potency (Table 2) [91]. Crystallographic studies (PDB ID: 5OEW) have demonstrated that **27** occupies the allosteric site in the GluA2 receptor in a manner that reproduces the interactions observed for two BPAM344 molecules bound simultaneously to symmetrical allosteric pockets [91]. Additionally, BPAM538 was also found to present modulatory effects at the kainate receptor subtype GluK1, most likely by stabilizing the dimer interface rather than altering domain closure, thus highlighting its potential as a versatile modulator with dual AMPA and kainate receptor activity. Electrophysiological recordings from HEK293 cells expressing homomeric GluK1(*Q*)_b_, GluK2(*VCQ*)_a_, or GluA2(*Q*)_i_ receptors confirmed that BPAM538 increased the amplitude of the peak response and delayed the onset of desensitization (Table 3) [93].

The ^11^C-radioisotope labeled BPAM538, [^11^C]AMPA-1905, has recently been developed as a novel PET ligand, enabling the visualization of AMPARs in the human brain; however, the authors indicated that further modifications to the chemical scaffold are needed in order to overcome the low binding potency and rapid brain washout of the ligand [95].

Drapier et al. further refined the dimeric ligand approach by designing structures that are capable of bivalently interacting with residues within the allosteric binding sites located on both sides of the AMPA-LBD–dimer interface (**28**, **29**) [94]. Their binding mode was confirmed by resolving the crystal structure of compound **28** in complex with the GluA2-LBD (PDB ID: 6FAZ), which validated its ability to interact with both allosteric binding sites on adjacent monomers, presumably leading to the complete inhibition of desensitization. The most active compound in this series, **29**, demonstrated an EC_50_ value of 1.4 nM—markedly surpassing the potency of its monomeric counterpart (BPAM344) and ranking among the most potent AMPAR-positive allosteric modulators reported to date (Table 3) [94].

Etsé et al. have proposed an innovative approach for the design of positive allosteric modulators involving the isosteric replacement of two nitrogen atoms in the thiadiazine ring with carbon atoms, which led to the development of thiochromane 1,1-dioxides [84]. This structural modification was intended to enhance the potential for distribution within the CNS. Among the synthesized derivatives, BPAM549 (**30**) demonstrated lower potency in vitro at AMPARs compared to its precursor, BPAM344. However, in vivo studies evaluating its BBB permeability revealed that **30** is efficiently absorbed from the gastrointestinal tract and effectively penetrates the BBB. X-ray analysis allowed for the identification of the (*R*)-**30** enantiomer as the preferred form for binding at the GluA2 receptor (PDB ID: 6ZYU), underscoring its potential for receptor-specific modulation [84].

In ongoing research efforts aimed at enhancing the effectiveness of AMPAR and KAR modulators, significant attention has been directed towards the development of novel 1,1-dioxide derivatives of 6-chloro-3,4-dihydro-2*H*-thieno [3,2-e]-1,2,4-thiadiazines. These studies emphasized the advantage of alkyl substituent incorporation, including the introduction of cyclopropyl and allyl groups at position 4 or 2,4-dialkyl- and 2,3,4-trialkyl-substitution [85]. An analog of BPAM344, BPAM395 (**31**), was identified as the most potent AMPA receptor potentiator within the described series (Table 2). Crystallographic analysis of **31** in complex with the GluA2 LBD (PDB ID: 8QEZ) confirmed its ability to establish interactions with key residues of the allosteric binding site, similar to those observed for BPAM344. BPAM279 (**32**)—a tricyclic thienyl analog of the clinically tested S18986—exhibited moderate in vitro potentiation effects on AMPA receptors, comparable to its precursor. However, in vivo studies demonstrated its efficacy as a cognitive enhancer in mice, with significant improvements in cognitive performance observed at a low oral dose of 1 mg/kg, suggesting that compound **32** possesses favorable pharmacokinetic properties, including effective gastrointestinal absorption and BBB penetration [85].

A structure-based approach using an X-ray complex of the GluA2 LBD with IDRA-21 allowed further analogs of IDRA-21 to be designed—in particular, compound **33,** with an improved pharmacological profile characterized by the presence of a furan ring in the benzothiadiazine core [96]. Compound **33** was found to express a significantly higher degree of potency in terms of enhancing glutamate-induced currents in both AMPA and KA receptors when compared with IDRA-21. In addition, both compounds displayed higher potency and efficacy at native receptors compared to recombinant systems, and in the latter, in contrast to IDRA-21, **33** showed a clear preference for AMPAR subtypes (Table 4) [97]. Studies of the hepatic metabolism of **33** using rat liver microsomes led to the identification of an unsaturated metabolite, **34**, which demonstrated biological activity comparable to that of **33** (in patch clamp assays, **33** enhanced KA-evoked currents by 358%, while that of the hepatic metabolite **34** was 323%). Furthermore, **34** exhibited enhanced chemical stability and, due to the absence of stereogenic centers, resolved the issue of rapid enantiomerization of **33** in aqueous solutions. In cerebral microdialysis studies on mice, **34** was able to permeate the BBB, leading to an augmentation in acetylcholine and serotonin levels within the hippocampus [98].

Due to the rapid enantiomerization in aqueous solution and hydrolysis in acidic environments observed for IDRA-21 and related compounds, Carozzo et al. developed a new derivative, **35** (Figure 4), combining the characteristic structural features of **33** and S18986 in order to obtain increased chemical stability while maintaining biological activity [99]. The effects of **35** and its enantiomers on AMPAR/KAR potentiation have been evaluated via a patch clamp assay in primary cerebellar neuron cultures, in which both **35** and IDRA-21 enhanced kainate-induced currents. In particular, (-)-(*R*)-**35** resulted in greater enhancement in currents induced by either kainic acid or (*S*)-5-fluorowillardiine than did (+)-(*S*)-**35**. These enantioselective differences in the activity of **35** can likely be attributed to different orientations in the ligand-binding domain and different interaction patterns in the receptor [99].

The agonist properties observed for some AMPAR PAMs, such as **22**, are believed to be related to bell-shaped dose–responses in pharmacological assays and increased risks of seizures. A structure-based design strategy to develop AMPAR PAMs with a minimized likelihood of inducing seizure-related side effects has led to the identification of new dihydropyrido-thiadiazine-2,2-dioxide analogs with minimal agonist effects, including TAK-137 (**36**) and TAK-653 (**37**) (Table 5) [100,101]. In a calcium flux assay and electrophysiological recordings in primary neurons, both compounds induced AMPAR-mediated responses in an agonist-dependent manner. Moreover, both compounds showed little subunit selectivity among recombinant GluA1-GluA4 AMPAR subtypes, including flip and flop isoforms [100,101]. According to the findings of crystallographic studies (PDB ID: 5ZG3 and 7F3O), the limited agonist activities of TAK-137 and TAK-653 can be attributed to the glutamate-dependent effect of steric interference at S750 located at the GluA2_o_ intradimer interface. Due to the change in the position of this amino acid in the receptor upon glutamate binding, the steric repulsion effect is particularly pronounced in the closed state of the channel [100,101].

In preclinical studies, TAK-137 induced brain-derived neurotrophic factor in neurons in rodents and potently improved cognition in both rats and monkeys, demonstrating a wide safety margin against seizures at the same time. Moreover, TAK-137 has exhibited encouraging levels of efficacy in various models of schizophrenia in rodents and non-human primates [102]. These findings suggest that TAK-137, with its broad therapeutic window and promising procognitive properties, has potential for use in the treatment of schizophrenia and other diseases associated with cognitive impairments. Additionally, both **36** and **37** have demonstrated antidepressant effects similar to those of ketamine but without psychotomimetic side effects, making them promising candidates as novel rapid antidepressants [103,104,105].

### 3.3. Sulfonamides and Related Dimeric Compounds

**Figure 5:**
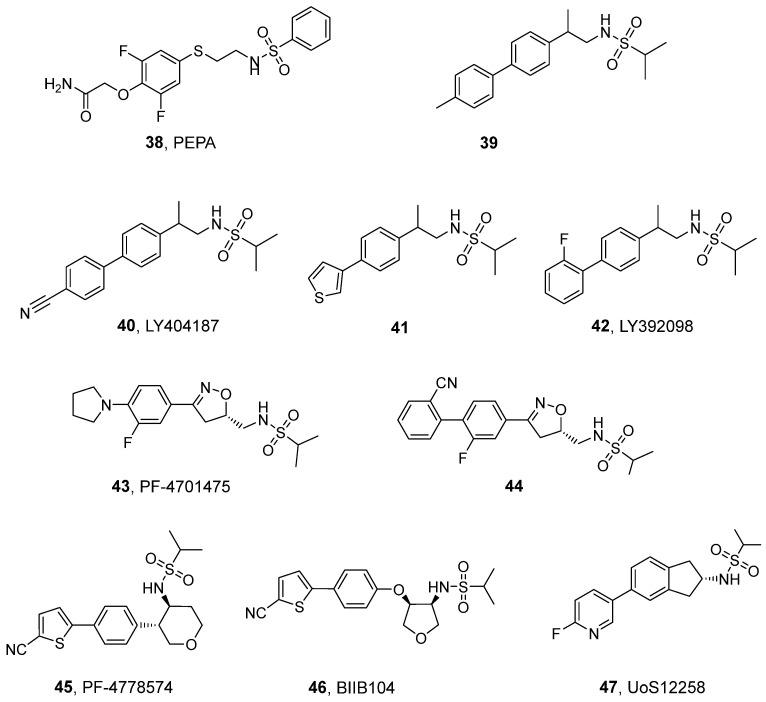
Structures of selected AMPAR PAMs with sulfonamide-based structures.

The sulfonamide-structured analogs described in this section represent the third major group among the chemotypes of positive allosteric modulators of AMPA receptors, which are structurally characterized by the presence of an alkyl-substituted sulfonamide group with a sulfur atom not incorporated in the ring system. One of the first compounds developed in this group is PEPA (**38**, Figure 5), which was found to be at least 100 times more potent for the amplification of glutamate responses at GluA3_o_ than aniracetam. PEPA preferentially potentiated flop isoforms (compared to flip variants) with a selectivity profile similar to aniracetam, in contrast to cyclothiazide, which is flip-selective [106]. As shown in X-ray studies of PEPA in complex with GluA2_o_ and GluA3_o_ (PDB ID 3M3L, 3M3F), the preference for the flop isoform can be explained by the formation of a hydrogen bond between the amide group in PEPA and the asparagine residue N754, which is replaced by serine in the flip variant [17].

In preclinical studies using the Morris water maze model, PEPA at doses lower than 10 mg/kg i.v. alleviated post-ischemic memory deficits in rats following unilateral middle cerebral artery occlusion, while aniracetam was ineffective under the same conditions [23]. In later reports, PEPA at a dose of 30 mg/kg i.p. was also found to facilitate the extinction of contextual fear memory in mice without any effect on the acquisition and consolidation of fear memory itself. This effect was shown to be mediated through the activation of AMPA receptors, mainly in the medial prefrontal cortex [107,108].

The first *N*-biaryl-2-propanesulfonamides **39**–**42** (Figure 5) were derived by modifying a methanesulfonamide lead structure. Among them, both **40** (LY404187) and **42** (LY392098) demonstrated a preference for GluA2_i_ and GluA4_i_ homomeric receptors over GluA1_i_ and GluA3_i_ (Table 6) and were several times more potent at the flip isoform of GluA2 than at the flop variant [109]. The preference for the flip variant can be explained by the formation of a direct hydrogen bond between the compound and the alternatively spliced Ser754 present in the flip form, as observed from the crystal structure of LY404187 in complex with GluA2_i_ (PDB ID: 3KGC) [14,17]. Extensive in vitro studies of LY404187 and LY392098 focused on AMPAR potentiation effects in electrophysiological recordings have been reported [110,111,112] (Table 7).

In preclinical in vivo studies, both LY392098 and LY404187 were shown to enhance cognitive function in a water maze performance test requiring working memory [111,112]. LY404187, with a minimum effective dose (MED) of 0.001 mg/kg p.o., reversed the memory deficit in an amnesic rat model of passive avoidance behavior [112]. Moreover, both compounds have been evaluated in behavioral models of depression [113]. LY392098 has demonstrated antidepressant-like effects in forced swim (with MED 0.5 mg/kg i.p.) and tail suspension (with MED 5 mg/kg i.p.) tests in mice and rats, reducing immobility time without affecting motor activity, indicating that its efficacy in the forced swim test is not related to motor stimulant action [114]. Similar findings have been reported for LY404187 [112], which reduced immobility in the test in both mice and rats, with MEDs of 0.05 and 0.025 mg/kg p.o., respectively. The *R*-isomer of LY404187 (LY451646) produced increased anxiety-like behavior in a mouse elevated zero maze test at doses higher than 3 mg/kg i.p. [115]. Moreover, at a dose of 3 mg/kg i.p., LY451646 has been shown to enhance the antidepressant-like effects of a selective serotonin reuptake inhibitor (i.e., citalopram) in the forced-swim test (FST) in mice while blocking its anxiolytic-like effects in the marble-burying test and elevated zero maze test. These results indicate that AMPAR neurotransmission may play opposite roles in anxiety and depression [116].

**Table 6:** Potentiation effects of selected sulfonamide-based PAMs at recombinant homomeric AMPA receptors.

Compound	EC_50_ [µM] (E_max_ [%])						
	GluA1_i_	GluA1_o_	GluA2_i_	GluA2_o_	GluA3_i_	GluA4_i_	GluA4_o_
LY404187 (**40**)	5.65 *^a,b^*	nd	0.15 *^a,b^*	1.44 *^a,b^*	1.66 *^a,b^*	0.21 *^a,b^* 1.0 *^c^* (120)	nd
LY392098 (**42**)	1.77 *^a,b^*	nd	0.22 *^a,b^*	0.55 *^a,b^*	1.89 *^a,b^*	0.20 *^a,b^* 1.2 *^c^* (120)	nd
PF-4778574 *^d,e^* (**45**)	nd	nd	0.045 (111)	0.090 (112)	nd	nd	nd
BIIB104 (**46**)	0.3 *^f^*	22*^f^*	0.024 *^d,g^* (124)	0.880 *^d,g^* (108)	nd	0.30 *^f^*	9.0 *^f^*
UoS12258 *^a,h^* (**47**)	10.0	nd	2.69 (101)	nd	6.76	2.24	nd

*^a^* effects on glutamate-induced (100 μM) response in HEK293 cells, intracellular calcium assay; *^b^* Ref. [109]; *^c^* effects on glutamate-induced (1 mM) current in HEK293 cells, voltage clamp method, Ref. [109]; *^d^* effects on (*S*)-AMPA-induced (32 μM) response in HEK293 cells, intracellular calcium assay; *^e^* Ref. [117]; *^f^* effects on glutamate-induced (10 mM) current in HEK293 cells, voltage clamp method, Ref. [7]; *^g^* Ref. [118]; *^h^* Ref. [119]; nd—not determined.

**Table 7:** Potentiation effects of sulfonamide analogs at native AMPA receptors, measured via electrophysiological recordings.

Compound	EC_50_ [nM]	E_max_
LY404187 (**40**)	1300 *^a^*	45.3-fold *^a^* 83% at 100 nM *^b^*
LY392098 (**42**)	1700 *^c^*	31.0-fold *^c^* 180% at 1µM *^b^*
PF-4701475 (**43**) *^d,e^*	123	147%
**44** *^d,e^*	<10	nd
PF-4778574 (**45**) *^d,f^*	919	162%
BIIB104 (**46**) *^d,g^*	310	110%
UoS12258 (**47**) *^h^*	7.94	nd

*^a^* effects on the glutamate-induced (100 μM) currents in isolated pyramidal PFC neurons, Ref. [112]; *^b^* effects on glutamate-induced (100 µM) currents in isolated rat cerebellar Purkinje neurons, Ref. [110]; *^c^* effects on the (*S*)-AMPA-induced (5 μM) currents in isolated pyramidal PFC neurons, Ref. [111]; *^d^* effects on the (*S*)-AMPA-mediated intracellular calcium concentration in mouse embryonic stem cell-derived neurons; *^e^* Ref. [120]; *^f^* Ref. [117]; *^g^* Ref. [118]; *^h^* effects on the AMPA-induced (30 μM) currents in rat hippocampal neurons, Ref. [119]; nd—not determined.

Given the promising results and high therapeutic potential of AMPA potentiators with sulfonamide structures, drug discovery research focusing on this chemical class has been particularly intensive. In a series of patents and articles, Estep et al. and Shaffer et al. described the potentiating effects on AMPARs among several classes of sulfonamide analogs based on HTS screening using a functional assay of mouse embryonic stem (mES) cell-derived neurons [117,118,120,121,122,123]. The assay results—supported by structure-based studies and physicochemical profile optimization—allowed for the identification of dihydroisoxazole compounds **43** (PF-4701475) and **44** (among others), which demonstrated high nanomolar potency (Table 7) and desirable in vitro ADME properties [120,124].

Other compounds that emerged from the mES screening, including the tetrahydropyran and tetrahydrofuran derivatives PF-4778574 (**45**) and BIIB104 (PF-04958242, pesampator, **46**) [117,118], have been examined in a series of in vitro and single-dose in vivo assays evaluating their AMPAR-mediated activities related to cognition and safety. Both compounds showed higher potentiating effects at the flip isoform of the GluA2 receptors compared to the flop variant (Table 6) [118]. The flip-selectivity of **46** was also observed in a study on flip/flop variants of GluA1 and GluA4 recombinant receptors [7]. Analysis of the X-ray structure of BIIB104 bound to the GluA2_o_ LBD (PDB ID: 4X48P) demonstrated a direct hydrogen bond between the ligand’s sulfonamide moiety and proline P494, while the cyano group was located in the vicinity of N754. It has been suggested that, in the flip variant, there is a small, flip-specific pocket at this site that contains the S754 side chain and promotes interactions with the cyano group of the ligand, contributing to the observed flip selectivity [118]. In electrophysiological recordings from recombinant AMPARs expressed in HEK293 cells, BIIB104 was found to completely eliminate desensitization of homomeric GluA1_i_ and GluA4_i_, slowing their deactivation by 4- to 5-fold; meanwhile, in the case of the corresponding receptors in flop isoforms, both effects were less pronounced [7].

To simulate the actions of PAMs on AMPA receptors located at synapses in different brain regions, Ishii et al. investigated the influence of BIIB104 on the potentiation and deactivation of AMPARs co-expressed with the auxiliary proteins stargazin/γ-2 and γ-8. Stargazin binds predominantly with synaptic AMPARs in cerebellar granule cells, where the enhanced signaling caused by PAMs may lead to side effects such as motor function impairment and Purkinje cell toxicity. On the other hand, in hippocampal CA1 pyramidal cell neurons, AMPARs form obligatory complexes with TARP γ-8; the modulation of these receptors is likely to be associated with procognitive effects. These studies have been extended through measurements of EPSCs modulated by **46** in acute cerebellar and hippocampal slices. The results showed that BIIB104 prolonged the deactivation of glutamate-evoked currents and the decay of synaptic currents to varying degrees, depending on the type of auxiliary protein in the composition of the receptor complex or the type of neuron. The most substantive results were noted for the AMPA–stargazin complex and the corresponding cerebellar granule cells [7].

Prior to in vivo pharmacology tests, **43**, **45**, and **46** were evaluated in pharmacokinetics studies in different species (rodents, dogs, and non-human primates) in order to optimize their dosage. The studies have confirmed the acceptable safety profiles of **43** and **46** for their preclinical progression [117,118,120]. However, studies in rats and dogs showed a dose-dependent toxicity of PF-4778574, inducing generalized convulsions at specific unbound brain concentrations. In non-human primates (nhp), PF-4778574 at a subcutaneous dose of 0.32 mg/kg resulted in movement-related tremors and ataxia, which was observable from 2 to 4 h after administration [117].

All three analogs were examined in an electrophysiological model of cognitive deficits in rats with schizophrenia based on pharmacologically provoked NMDAR hypofunction. This assay evaluates a compound’s ability to affect monosynaptic projections from the hippocampus/subiculum to the medial prefrontal cortex, which contributes to working memory. In the test, compounds **43**, **45**, and **46** significantly reversed the disruptive effects of the NMDAR antagonist MK-801 on both cortical electroencephalography (EEG) and paired-pulse facilitation (PPF) at doses of 0.003 mg/kg, 0.1 mg/kg, and 0.01 mg/kg i.v., respectively, indicating the ability of these compounds to attenuate cognitive deficits in patients with schizophrenia [117,118,120]. In addition, PF-4778574 and BIIB104 (at doses of 0.01 mg/kg and 0.0032−0.032 mg/kg s.c., respectively) decreased the ketamine-mediated impairment of spatial working memory in non-human primates [117,118]. PF-4778574 has also been found to rapidly alleviate depression-like behaviors in mice [125]. Co-treatment with low doses of the compound (0.5 mg/kg i.p.) and the glycogen synthase kinase 3β (GSK3β) inhibitor (10 mg/kg) induced synergistic antidepressant-like effects in the FST in mice [126]. In experimental murine models of multiple sclerosis, PF-4778574 led to notable improvements in clinical indices of autoimmune encephalomyelitis, reduced neuronal loss, and had positive effects on maintaining myelin integrity. These outcomes attenuate the clinical disability of PF-4778574 in experimental models of multiple sclerosis following both prophylactic and therapeutic administration [127].

On the other hand, the promising results of preclinical and safety studies on BIIB104 have led to the classification of this compound as a clinical candidate, consequently subjecting it to further investigation in human clinical trials (see Section 4).

The ^11^C-labeled BIIB104 has recently been developed to measure the brain penetrance and kinetics of the compound. PET studies in rhesus monkeys demonstrated substantial uptake of the radioligand into the brain, with little variability in its distribution across different regions [128]. Another radioligand—labeled with ^14^C in the nitrile moiety—has been developed and used to evaluate the metabolism of BIIB104. The study revealed the unexpected metabolism of [nitrile-^14^C]BIIB104 and the formation of [^14^C]cyanide from 2-cyanothiophene as a major metabolite in rat liver microsomes [129].

The development of a new series of indane-propylsulfonamide derivatives yielded UoS12258 (GSK729327, **47**) [130]. The results of an intracellular Ca^2+^ influx assay using various subtypes of homomeric human and rat AMPARs revealed that UoS12258 exhibits neither subunit nor species specificity (Table 6) [119]. In rat hippocampal neurons subjected to whole-cell voltage clamp electrophysiology, the application of 30 μM AMPA in the presence of UoS12258 produced inward currents with larger peak amplitudes and reduced desensitization while slowing the deactivation process. Furthermore, the compound at a concentration of 100 µM did not affect NMDA or kainate receptor-mediated currents [119].

UoS12258 has been investigated in behavioral models of cognition and was found to improve performance in novel object recognition tests in rats after both acute (0.3 mg/kg p.o.) and sub-chronic (with an MED of 0.03 mg/kg p.o.) doses [119]. Additionally, **47** prevented scopolamine-induced impairment of a passive avoidance response and improved aged-induced deficits in learning and retention in the water maze test in rats; however, these effects were observed at doses higher than those in the NOR test [119]. During preclinical screening of anticonvulsant activity, **47** was found to significantly reduce the seizure threshold at doses greater than 10 mg/kg in the maximal electroshock seizure threshold test (MEST) in rats [119]. A detailed preclinical evaluation in rats, dogs, and monkeys revealed good CNS penetration and favorable pharmacokinetic parameters of UoS12258 [130], which, combined with the results of studies using behavioral cognitive models, led to the qualification of this compound for clinical trials in terms of diseases involving cognitive impairments (see Section 4).

**Figure 6:**
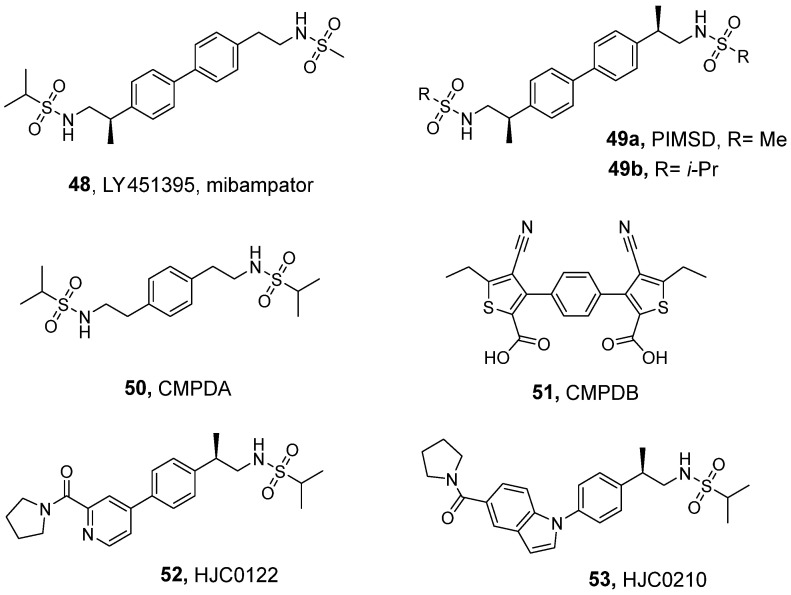
Structures of selected sulfonamide-based AMPAR PAMs.

Further optimization of the biarylsulfonamide scaffold led to the discovery of a number of dimeric compounds [131], including LY451395 (mibampator, **48**, Figure 6), which contains two sulfonamide moieties linked by a biaryl bridge in its structure. Among others, **48** has been examined using electrophysiological techniques in recombinant homomeric AMPA receptors (flip and flop splice variants) expressed in HEK293 cells, where it demonstrated submicromolar potency (Table 8) while exhibiting low selectivity between AMPARs with distinct subunit compositions [7]. Moreover, **48** eliminated the desensitization of homomeric GluA1_i_ and was less efficacious on GluA1_o_, consistent with the results obtained for LY404187 and LY392098 (which were also more potent at flip isoforms) [7]. The effects of **48** on receptor deactivation have been investigated in outside-out patches pulled from HEK293T/17 cells expressing homomeric GluA1 and GluA4 flip/flop isoforms as well as heteromeric GluA1/GluA2 and GluA2/GluA4 receptors. This heteromeric subunit composition is believed to occur in synapses located between hippocampal Schaffer collaterals and CA1 pyramidal neurons, as well as between cerebellar mossy fibers and granule cells. The study demonstrated that LY451395 slows the deactivation rate of both homomeric and heteromeric AMPARs, exhibiting neither splice variants nor subunit selectivity [7]. Furthermore, it has been shown—through both the patch clamp and calcium influx assays—that LY451395 can activate AMPARs in primary neurons in the absence of an agonist, suggesting agonistic properties at physiological AMPA receptors [132]. Interestingly, in electrophysiological studies using brain slices from adult rats and non-human primates, **48** showed more pronounced effects in rat native AMPARs compared with those from nhp [133]. The crystal structure of the complex of **48** with the GluA2_o_ LBD (PDB ID: 5YBG) revealed one dimeric bivalent molecule of the ligand, forming interactions with the residues of two adjacent protomers at the same time; in particular, two sulfonamide moieties of **48** interacted via hydrogen bonds with the backbone atoms of both prolines P515, while the biphenyl core formed van der Waals contacts with this residue [132].

Based on the concept of bivalent ligands, Kaae et al. reported another series of symmetrical dimeric AMPAR PAM ligands with submicromolar potency [134]. Some of these analogs have displayed potency three orders of magnitude higher when compared to their monomeric counterparts. In detailed in vitro studies on individual AMPAR subtypes, the lead compound PIMSD (**49a**) was found to show only small differences in potency at the homomeric receptors GluA1_i_-4_i_ and presented no flip/flop selectivity between GluA2_i_ and GluA2_o_ (Table 8). The structure of PIMSD in complex with the GluA2 LBD (PDB ID: 3BBR) demonstrated that only one molecule binds to the dimer interface, with two sulfonamide moieties occupying CTZ-binding sites, while the central biphenyl system plays the role of a bridge between two allosteric binding sites located at both GluA2 protomers [134]. In animal studies using a mouse model of scopolamine-induced impairment of spatial navigation, **49a** was found to partially reverse the impairment caused by scopolamine at a dose of 3 mg/kg s.c. [136].

Timm et al. investigated the structural and functional properties of two PAMs of AMPAR: CMPDA (**50**) and CMPDB (**51**) [135]. The effects of both compounds on deactivation and desensitization were examined using short (1 ms) and long (500 ms) pulses of glutamate (10 mM), respectively, measuring the responses from HEK293 cells expressing recombinant rat GluA2 flip/flop receptors. The results indicated that **50** was clearly flop-selective, while **51** was more flip-selective in attenuating receptor deactivation. On the other hand, **50** was shown to modulate receptor desensitization of both the flip and flop isoforms of GluA2, similarly to CX614, whereas **51** was more effective in blocking flip isoform receptor desensitization [135]. Analysis of the X-ray structures of both analogs bound to the GluA2_o_ LBD (PDB ID: 3RNN, 3RN8) revealed that in both cases, a single molecule of the compound binds at the dimer interface, with the interaction pattern being similar to the binding modes of CTZ and CX614 [135,137]. The unique profile of **51**—which enhances excitatory signaling while maintaining isoform selectivity—makes it an interesting candidate for consideration in further drug discovery studies.

A series of highly potent AMPAR PAMs containing a sulfonamide moiety were patented by Zhou et al. [138]. The structures of these compounds contain an arylpropylsulfonamide moiety from one side, which is linked through the aromatic ring with a benzamide, sulfonamide, lactam, or sulfone scaffold from the opposite side. This type of structure allowed for bivalent binding to the receptor, resulting in high nanomolar (or even picomolar) potency of compounds **52** and **53** (EC_50_ = 6.53 nM, E_max_ = 170%, and EC_50_ = 0.06 nM, E_max_ = 157.5%, respectively; tested via a calcium influx assay using DIV9 dissociated rat forebrain neurons) [138]. In an in vivo study, **52** was shown to prevent and reverse morphine tolerance and physical dependence in mice (at a 10 mg/kg dose) in the tail-flick test, opening a new direction for preventing opioid drug tolerance and dependence [27].

### 3.4. Trifluoromethylpyrazoles

**Figure 7:**
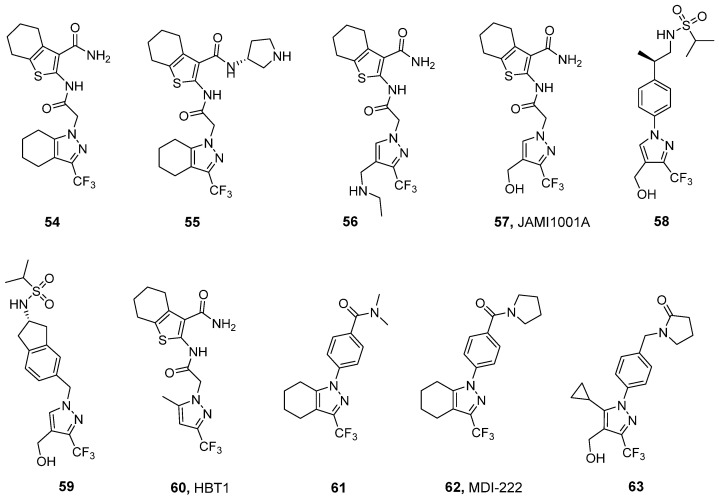
Structures of selected AMPAR PAMs based on a trifluoromethylpyrazole structure.

The series of compounds based on trifluoromethyl pyrazole scaffolds constitutes a novel AMPAR PAM chemotype that has been intensively studied over the past decade. Hit-to-lead and lead optimization studies in this series (**54–59**, Figure 7) have been reported by researchers from Merck Laboratories in a series of papers [139,140,141,142]. The hit compound **54** was identified via high-throughput screening using a calcium influx assay with GluA1_i_ overexpressed in HEK293 cells. The X-ray structure of **54** in complex with GluA2_i_ (PDB ID: 3O28) revealed two key structural elements—namely, a trifluoromethyl moiety and a tetrahydrobenzothiophene group—which contribute to essential hydrophobic contacts with the receptor. To address the problems related to the low solubility (<1 mg/L) and poor metabolic stability of **54**, modifications to the tetrahydrobenzothiophene, tetrahydroindazole, and amide regions were performed. The resulting analog **55** demonstrated improved physicochemical properties and microsomal stability; however, it suffered from low oral bioavailability (F = 3.8%) [139].

Further optimization led to **56**, in which the tetrahydroindazole group was replaced by a secondary amine in the trifluoromethyl pyrazole region. Compound **56** demonstrated significantly improved oral bioavailability (F = 43%) while maintaining receptor potency (Table 9) and reasonable pharmacokinetics [140]. The hydroxyl-containing analog of **56**, JAMI1001A (**57**)—in a patch clamp assay using outside-out membrane patches from HEK293 cells transiently expressing the flip and flop isoforms of GluA2—turned out to modulate both the channel desensitization and deactivation of both isoforms [142]. This modulation profile—namely, a lack of isoform selectivity—is distinctly different from that of other known PAMs, such as CTZ or CX614. Analysis of the X-ray structure of **57** in complex with the GluA2_i_ LBD (PDB ID: 4FAT) revealed that **57** binds with one molecule at the dimer interface of the AMPA receptor. The trifluoromethyl group at the pyrazole ring and the cycloalkane ring of tetrahydrobenzothiophene primarily mediate hydrophobic interactions at symmetrical subsites on adjacent monomers (similar to the norbornenyl group of CTZ), while an amide linker is located at the center of the dimer interface. In addition, hydrogen bonds are formed on two sides of the molecule: one between the carboxamide group on the cycloalkane ring and S497 and another between the hydroxyl group at the 4-position of the pyrazole ring and three residues: S729, P494, and the isoform-specific S754. It should be noted, however, that in in silico studies of GluA2 with S754 mutated to asparagine, an interaction between N754 and the ligand’s hydroxyl group was also predicted, which may explain the observed lack of isoform selectivity [142].

To improve its bioavailability, hybridization of **57** with **40** was performed, resulting in dimeric analog **58** [141]. However, despite the good metabolic stability and permeability observed in the CaCo-2 assay, compound **58** showed poor oral bioavailability (F = 3.2%). Incorporating an indane moiety as a conformational constraint led to compound **59** ((*S*)-enantiomer), which achieved significantly better bioavailability (F = 94%) [141].

The increased risk of seizure induction has been linked with agonist properties observed for certain AMPAR PAMs, such as **22**, LY451646 (the (*R*)-enantiomer of **40**), and **48**, and bell-shaped dose–response relationships in pharmacological assays. Avoiding this type of activity has, therefore, become a starting point in the search for subsequent PAMs, leading to the development of a new AMPAR potentiator: HBT1 (**60**) [132]. This compound activates AMPARs in an agonist-dependent manner, demonstrating reasonable potency at the native receptors in primary neurons and the GluA1_i_ receptors expressed in CHO cells (Table 9). Furthermore, it exhibited a weak agonistic effect in a Ca^2+^ influx assay, a patch clamp study, and a BDNF assay in primary neurons, as well as lower risk of a bell-shaped response in BDNF production [132]. Despite these promising properties, **60** was found to be unsuitable for further in vivo research due to its poor ADME profile.

GlaxoSmithKline’s research focused on identifying AMPAR PAM compounds has entailed the utilization of a high-throughput screening approach, employing a calcium influx assay on a human recombinant GluA2_i_ cell line to facilitate the selection of promising molecules. The hit compound **61** demonstrated desirable selectivity against a variety of ion channels, receptors, and enzymes, in addition to a favorable P450 profile [143,145]. The subsequent hit optimization workflow, supported by crystallography studies, concentrated on enhancing the solubility and metabolic stability of the compound. Among others, replacement of the dimethyl amide group with cyclic pyrrolidine resulted in MDI-222 (**62**, Table 9), which presented higher potency and significantly improved metabolic stability in both rat and human microsomes [143]. In recombinant systems, **62** demonstrated comparable AMPAR-specific potency at all four homomeric human AMPAR subtypes (pEC_50_ in the range of 4.5 to 5.4) and no marked human-to-rat species differences [144]. Furthermore, **62** potentiated AMPAR-mediated currents in cultured rat hippocampal neurons and was found to enhance electrically evoked AMPAR-mediated synaptic transmission in anesthetized rats at 10 mg/kg i.v. In animal studies, MDI-222 resulted in enhanced cognitive performance in the novel object recognition test in rats, with MEDs of 0.3 mg/kg p.o. after single-dose administration and 0.1 mg/kg p.o. following sub-chronic dosing. It was also effective in reversing scopolamine-induced memory impairment in a passive avoidance test in rats, with an MED of 10 mg/kg p.o. In addition, the compound did not show proconvulsant properties in the MEST assay in rats at a dose of 30 mg/kg p.o., resulting in a large (1000-fold) therapeutic window between the plasma concentrations required for efficacy and those associated with convulsions and other side effects [144]. In contrast to UoS12258, **62** did not demonstrate convulsant activity in 4-week GLP Preclinical Safety and Toxicity studies in either rats or dogs treated with the highest doses, showing a much safer profile compared to UoS12258 [119,144]. Nevertheless, despite its favorable preclinical profile, further development of **62** was terminated due to solubility issues, high protein binding, and activation of the pregnane X receptor [144].

In 2021, Ward et al. patented a series of new AMPAR PAMs with structures based on a trifluoromethylpyrazole fragment linked with an *N*-benzylpyrrolidin-2-one core (**63**) [146]. The potentiating effects of the compounds on glutamate responses at a test concentration of 40 µM were demonstrated in a calcium ion influx assay in HEK293 cell lines expressing homomeric hGluA2_i_ receptors. The procognitive properties of the selected analogs were proven in the NOR test in rats, with minimum effective doses of the compounds being less than 10 mg/kg following oral administration.

### 3.5. Recently Developed Bivalent Ligands

New classes of bivalent compounds have recently been explored and reported in a series of articles [147,148,149,150], driven by the observation that such AMPAR modulators exhibit significantly higher potency compared to their monomeric counterparts [134]. This enhanced activity has been linked to the U-shaped configuration of the AMPA receptor’s allosteric binding site, which allows bivalent ligands to bind more efficiently [151].

**Figure 8:**
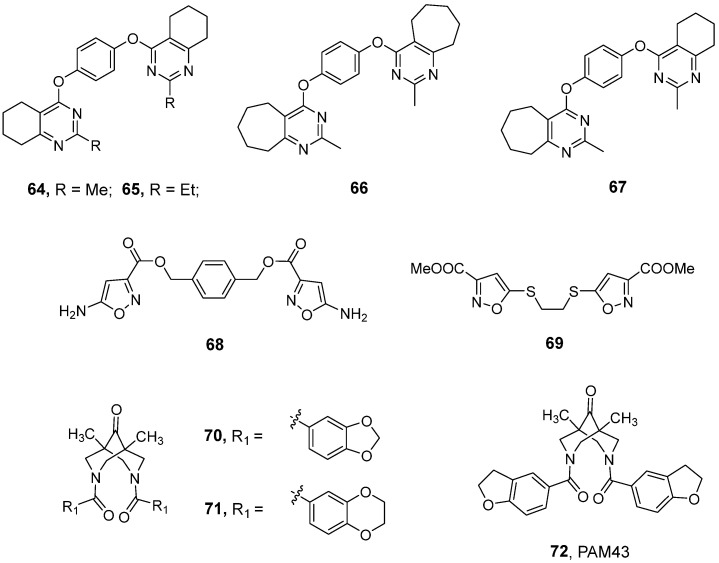
Structures of selected dimeric AMPAR PAMs.

Nazarova et al. described a series of dimeric compounds featuring two bulky pyrimidine fragments of tetrahydroquinazoline substituted with alkyl groups and connected by an oxygen-containing *p*-dioxyphenylene linker (Figure 8) [148]. The effects of the tested compounds on the potentiation of AMPA receptors have been investigated in isolated rat cerebellum Purkinje neurons using a kainate, which induces AMPA receptor-mediated currents while evoking relatively low receptor desensitization (Table 10). Compound **64** showed the highest positive modulating effect, with a 70% increase in kainate-induced current at a 1 nM concentration, while the ethyl-substituted analog **65** displayed lower potency [148]. Observations from docking studies suggested that **64** efficiently filled the symmetric subsites of the binding pocket and that the volume of ligand exposed to solvent was minimal, while exposure of 2-ethyl groups in **65** (or larger substituents in similar structures) led to a decrease in potency [148].

In order to investigate the relationships between bis(pyrimidine) analogs and their AMPAR-modulating effects, a broader series of substituted bis(pyrimidines) with symmetric and non-symmetric structures (**66**, **67**) was reported by Sedenkova et al. [150]. In patch clamp experiments on rat cerebellar Purkinje cells, most of the compounds demonstrated a bell-shaped concentration-dependent potentiation of AMPA receptor currents within a wide range of concentrations (10^−12^–10^−6^ M). The best results were found for compound **67**, which potentiated kainate-induced currents by up to 77% and, in contrast to **64**, did not act as a negative modulator at higher concentrations (Table 10). In addition to electrophysiological tests, physicochemical prediction and ADMET studies have been performed, the results of which confirmed that **67** is an acceptable candidate lead compound for the initial stage of ligand development [150].

Molecular design based on the results of previously reported pharmacophore and QSAR models [152], supported by molecular docking and MD simulations, led to the development of a series of bis(5-aminoisoxazoles) by Vasilenko et al. [153]. Among them, compound **68**—featuring a benzyl ester linker—emerged as the most promising (Figure 8). Electrophysiological measurements demonstrated its ability to potentiate kainate-induced AMPA receptor currents over a broad concentration range (10^−12^–10^−6^ M), with maximum potentiation at 10^−11^ M—comparable or superior to some of the most potent known PAMs and higher than that of CTZ [153]. Of the many other reported derivatives containing various linkers in the bis(isoxazole) scaffold, the only compound featuring a dithiol linker (**69**) has demonstrated remarkable AMPAR potentiation ability. In contrast, analogs with diamine and hydroquinone linkers act as moderate negative modulators [151].

**Table 10:** Potentiation effects of selected dimeric and tricyclic compounds on the kainate-induced (20 μΜ) AMPA receptor currents in rat cerebellum Purkinje cells, measured via the patch clamp method (adapted from Ref. [150]).

Compound	Number of Neurons	Potentiation Effects [%] Observed at a Specific Modulator Concentration (Control 100%)						Ref.
		10^−12^	10^−11^	10^−10^	10^−9^	10^−8^	10^−7^	
**64**	7	108	132	143	170	123	85	[150]
**67**	5	105	138	149	177	163	155	[150]
**68**	4	141	172	152	144	129	113	[153]
**69**	3	100	139	177	163	146	141	[151]
**72**	13	100	122	152	162	145	136	[154]
**73a**	3-6	143	129	117	122	109	110	[155]
**73b**	6	-	118	124	132	125	119	[156]
**74a**	7	111	141	152	162	148	135	[157]
**74b**	7	89	87	84	75	68	61	[157]

Expanding the scope of structural diversity, dimeric ligands with a rigid bicyclic spacer (3,7-diaza-bicyclo[3.3.1]nonan-9-one) have been designed by Lavrov et al. (Figure 8) [158]. This rigid spacer ensures optimal spacing between active fragments, facilitating their binding to symmetrical subsites of the allosteric binding pocket. On the basis of docking simulations, the authors selected lead structure **70** with two piperonyl moieties, which was shown to increase kainate-induced currents in Purkinje cells by 110% at 1 nM [147]. Analog **71** exhibited similar effects but was 10 times less potent, with maximum potentiation at 10 nM (80%) [158]. A further increase in its concentration led to a drop in the potentiating effect, demonstrating the bell-shaped dependence of AMPA potentiation. Furthermore, a docking study of compounds **70** and **71** showed that the active compounds presumably adopt a CTZ-like binding mode, with two ligand molecules interacting with symmetric subsites rather than one molecule occupying both subsites. In behavioral experiments, both **70** and **71** demonstrated anti-amnestic properties, as evidenced in scopolamine-induced amnesia and maximal electroshock models in rats [158].

Another interesting symmetric analog based on a 3,7-diaza-bicyclo[3.3.1]nonan-9-one core, containing two 2,3-dihydrobenzofuran groups—called PAM43 (**72**)—has been reported by Vyunova et al. [154]. In electrophysiological studies of rat Purkinje cells, PAM43 showed an ability to potentiate AMPA currents in a concentration-dependent manner (Table 10), reaching a maximum effect at 1 nM (62% increase in current). In order to characterize the specific binding mode of the ligand, synthesis of tritium-labeled **72** was carried out [154]. A study using [^3^H]PAM43 revealed the existence of at least two different binding sites in the receptor for this allosteric modulator. While one site is glutamate-dependent and characterized by a higher affinity, the other is glutamate-independent and characterized by a lower affinity for PAM43 [154,159]. In preclinical studies, PAM43 was found to stimulate memory and cognitive performance in rats in a dose range of 0.05–0.5 mg/kg [160,161].

**Figure 9:**
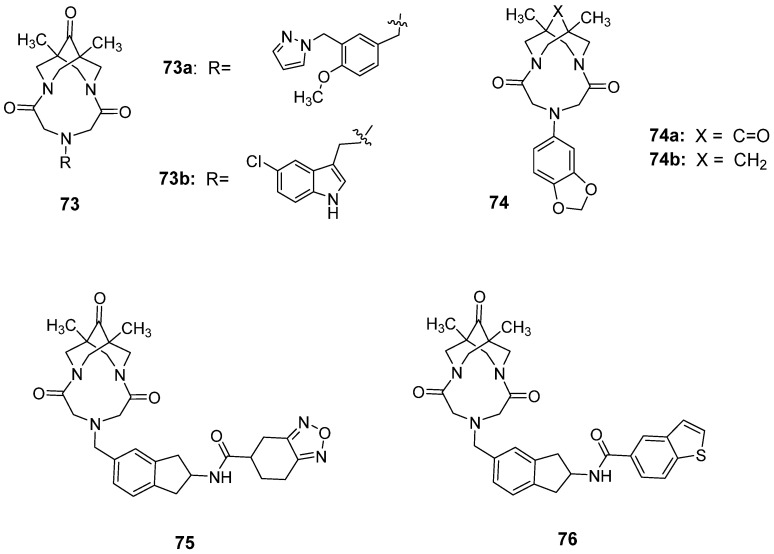
Structures of compounds with non-symmetric tricyclic scaffolds.

Lavrov et al. presented a series of compounds based on a non-symmetric tricyclic scaffold with the general formula **73** (Figure 9) [155]. The authors reported only one compound (**73a**), which exhibited the maximum potentiation of the kainate-induced currents in the patch clamp assay at a concentration of 10^−12^ M. A subsequent group of analogs with non-symmetric scaffolds was developed based on the tricyclic cage moiety and (un)substituted indole or 1,3-benzodioxole in the *N*-6 position [156]. Among the four compounds, only the chloroindole derivative **73b** demonstrated concentration-dependent potentiation of AMPA receptor currents [156]. Interestingly, in the case of two structurally similar bispidine derivatives (**74a** and **74b**), an opposite AMPAR modulation profile was observed. Compound **74a** (possessing a carbonyl group) acted as a positive allosteric modulator, while its analog **74b** (lacking this group) behaved as a negative modulator (Table 10). These findings underscore the profound influence of subtle structural changes on the receptor modulation ability of compounds [157].

Golubeva et al. reported more expanded analogs with indane fragments introduced into tricyclic scaffolds (**75**, **76**) [159]. Radioligand binding studies using [^3^H]PAM43 revealed that compounds **75** and **76**, rather than displacing the radioligand, increased its binding. This effect was particularly evident in the case of **75**, which, in the concentration range of 0.1–10 nM, potentiated the binding of [^3^H]PAM43 by almost two-fold. Based on this observation, the authors hypothesized that compounds **75** and **76** may not interact with the same binding sites of PAM43 but presumably bind to other specific sites on AMPAR, resulting in synergistic facilitation of PAM43 binding [159].

### 3.6. Miscellaneous Chemotypes

Several new miscellaneous chemotypes of AMPAR PAMs have been described in patent applications published during the period from 2020 to 2022.

**Figure 10:**
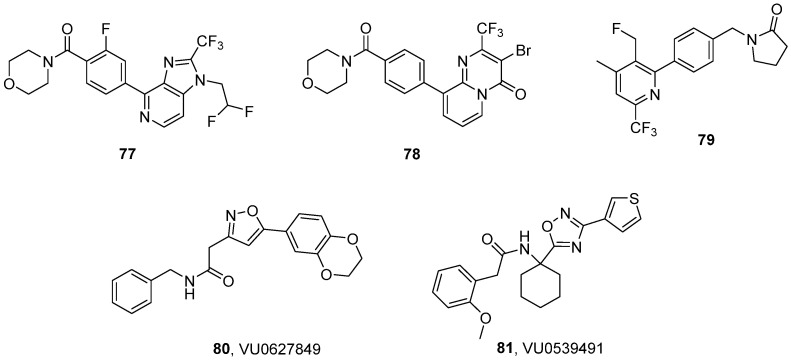
Structures of AMPAR PAMs with miscellaneous chemotypes.

Takeda Pharmaceuticals has patented several series of compounds with structures based on condensed heterocyclic cores, including benzimidazole, imidazopyridine (e.g., **77**, Figure 10), pyridopyrimidine-4-one (e.g., **78**), and pyrazinopyrimidine-4-one [162,163]. Supporting in vitro data from a calcium ion influx assay in CHO-K1 cell lines expressing hGluA1_i_ in complex with stargazin demonstrated the potentiating effects of the analogs on the glutamate responses at a test concentration of 30 µM. In vivo data from the NOR test in rats for selected compounds were also presented. Among them, compound **78** showed a significant improving effect on cognition function at a dose of 1 mg/kg p.o. [163].

In continuation of their research on trifluoromethylpyrazole analogs, Ward et al. recently developed a series of compounds with a broad range of structural modifications, including trifluoromethylpyridyl derivatives linked with pyrrolidin-2-one (**79**), morpholino-3-one, or oxazepanone groups [164]. The potentiating effects of these compounds on glutamate responses at a test concentration of 10 µM were demonstrated in a calcium ion influx assay in HEK293 cell lines expressing homomeric hGluA2_i_ receptors. In behavioral studies, the selected analogs showed procognitive effects in the NOR test after oral administration in rats (with an MED of less than 10 mg/kg) and did not present any proconvulsant activity in the maximum electroshock threshold test at doses up to 50 times higher than the MED found in the NOR test. Furthermore, the compounds described in the patent were—according to the authors—characterized by favorable drug metabolism and pharmacokinetic profiles, presenting an in vitro intrinsic clearance of less than 100 µL/min/kg in rat and human microsomes, as well as low human P-glycoprotein efflux liability.

Azumaya et al. have recently reported an innovative method based on high-throughput screening, allowing for the identification of positive and negative AMPAR allosteric modulators that act selectively on specific GluA2-auxiliary subunit complexes [165]. Using this approach, the researchers identified a compound with a novel AMPAR PAM chemotype featuring a disubstituted isoxazole-based motif (VU0627849, **80**, Figure 10). The compound displayed activity at concentrations of 2.5 μM and selectively potentiated the GluA2–stargazin and GluA2–CNIH3 (cornichon homolog 3) complexes while showing minimal activity on GluA2 receptors lacking auxiliary proteins. Interestingly, another hit compound, VU0539491 (**81**), showed different profiles depending on the type of AMPAR–auxiliary subunit complex: it behaved as a PAM in the GluA2(Q)–GSG1L (germ cell-specific gene 1-like protein) complex but presented NAM activity in the GluA2(R)–CNIH3 and GluA2(Q) complexes [165].

### 3.7. Con-Ikot-Ikot and ConA

While the majority of known PAMs are small molecules that target well-defined allosteric sites, a number of macromolecular compounds have been identified that modulate receptors through alternative binding sites and mechanisms of action. Conotoxins constitute an extensive group of highly potent peptide toxins that are discharged by predatory marine cone snails (genus *Conus*), which are prevalent in tropical marine ecosystems. Conotoxins act to rapidly paralyze the target prey by affecting voltage- or ligand-gated membrane ion channels, as well as G protein-coupled receptors (GPCRs) and neurotransmitter transporters [166]. To date, con-icot-icot is the only conotoxin identified that shows selectivity and high affinity toward AMPA receptors (EC_50_ of 5 nM), with a mechanism of action consisting of blockade of AMPA receptor desensitization and a weak effect on open channel stabilization [167]. Furthermore, con-icot-icot demonstrates a unique binding mode, immobilizing all four ligand-binding domains of the tetramer. This interaction functions as a molecular ‘four-legged clamp’, effectively maintaining the AMPA receptor channel in an open conformation [20].

Concanavalin A (ConA)—an exogenous lectin derived from mung bean extract—has been shown to irreversibly potentiate AMPA and kainate receptor currents by binding to *N*-glycosylated carbohydrate moieties on these receptors, with a higher selectivity for kainate receptors [168]. The binding sites for ConA and other lectins are localized within the extracellular regions of the amino-terminal domain and ligand-binding domain. Notably, receptor potentiation is contingent on the conformational state of the LBD, as preincubation with ConA before glutamate application has been shown to lead to a more substantial reduction in receptor desensitization, compared to its co-application with glutamate [169]. Furthermore, this preincubation effect was attenuated when partial agonists were used, suggesting a mechanism that is dependent on full receptor activation. The proposed mechanism underlying this modulation indicates that ConA inhibits kainate receptor desensitization and extends receptor activation, potentially by functioning as a structural spacer between the ATD and LBD [170]. In addition to plant-derived lectins, endogenous mammalian β-galactoside-binding lectins—known as galectins—have also been implicated in modulating AMPA and kainate receptor functions. A study conducted by Copits et al. demonstrated that human galectin-1 and eel-derived congerin-1 attenuate kainate receptor desensitization in dorsal root ganglion (DRG) neurons, thereby suggesting a potential role for galectins as endogenous positive allosteric modulators of receptor activity [171].

## 4. Clinical Relevance and Therapeutic Applications

In recent decades, a considerable number of clinical trials have been conducted to investigate the potential of AMPAR PAMs for the treatment of various CNS diseases, including depression, schizophrenia, attention deficit hyperactivity disorder, Alzheimer’s disease, and fragile X syndrome (see Table 11 for a summary). A majority of these trials have been unsuccessful in Phase II, with some being discontinued prematurely due to concerns regarding adverse events. Despite these setbacks, encouraging results have emerged for certain compounds.

A notable proportion of the extant clinical studies has focused on the field of CX compounds developed by RespireRx Pharmaceuticals (formerly Cortex Pharmaceuticals). CX516 ampakine (**5**, ampalex) has undergone extensive clinical trials targeting various cognitive domains. These include delayed recall and mild cognitive impairment in elderly patients, cognitive function and behavior in patients with fragile X chromosome syndrome, and cognitive impairment associated with schizophrenia [172,173,174]; however, CX516 demonstrated no efficacy in the clinical setting, likely due to its low potency and poor half-life [174].

Preclinical studies on farampator (CX691, ORG-24448, **7**) have revealed its promising potential for treating cognitive deficits in schizophrenia and AD patients. Phase I clinical trials investigating the acute effects of farampators on memory have revealed their potential impacts on memory and cognitive processing in healthy elderly volunteers. The administration of 500 mg of compound **7** resulted in a significant enhancement in short-term memory; concomitantly, however, it also exerted a negative influence on the subject’s episodic memory [175]. The compound had progressed to Phase II clinical trials as an adjunct to therapies with atypical antipsychotics in patients with schizophrenia; however, the study was withdrawn at the sponsor’s request. A clinical investigation of **7** for the treatment of depression was also terminated due to concerns about adverse events (including decreased white blood cell and absolute neutrophil counts).

**Table 11:** AMPAR PAMs subjected to clinical trials [172].

Intervention	Condition	Stage of Development Status	Reference
CX516, ampalex (**5**)	Schizophrenia	Phase II/Phase III Completed (2007)	[176], NCT00235352
	Fragile X syndrome/autism	Phase II Completed (2005)	[174], NCT00054730
	Mild cognitive impairment	Phase II Completed (2004)	[173], NCT00040443
	Alzheimer’s disease/dementia	Phase II Completed (2005)	NCT00001662
CX691, farampator (**7**)	Major depressive disorder	Phase II Terminated (2007)	NCT00113022
	Cognitive deficits in schizophrenia	Phase II Withdrawn (2009)	[175], NCT00425815
CX717 (**8**)	Attention deficit hyperactivity disorder	Phase II Completed (2006)	NCT03375021
CX1739 (**9**)	Opiate-induced respiratory depression	Phase II Uknown status (2016)	NCT02735629
ORG-26576 (**10**)	Major depressive disorder	Phase II Completed (2008)	[177], NCT00610649
	Attention deficit hyperactivity disorder	Phase II Completed (2009)	[178], NCT00610441
S47445, CX1632, turlampator (**18**)	Alzheimer’s disease	Phase II Completed (2017)	[179], NCT02626572
	Major depressive disorder	Phase II Completed (2017)	NCT02805439
S18986 (**22**)	Mild cognitive impairment	Phase II Terminated (2006)	[180], NCT00202540
TAK-653, osavampator (**37**)	Major depressive disorder	Phase II Completed (2024)	[181], NCT05203341
	Major depressive disorder	Phase III Recruiting	NCT06786624
BIIB104 (**46**)	Hearing loss	Phase I Completed (2013)	[182], NCT01518920
	Ketamine-induced cognitive impairment	Phase I Completed (2014)	[183], NCT01749098
	Cognitive impairment in schizophrenia	Phase II Completed (2022)	[184], NCT03745820
UoS12258, GSK729327 (**47**)	Schizophrenia	Phase I Completed (2009)	NCT00448890
LY451395, mibampator (**48**)	Alzheimer’s disease	Phase II Completed (2003)	NCT00051909
	Aggression and agitation associated with AD	Phase II Completed (2011)	[185], NCT00843518

Recent studies on the tolerability and pharmacokinetics of CX717 (**8**) have revealed its safety in both healthy men and elderly subjects of both genders over a wide dose range (25–1600 mg). The adverse effects were predominantly mild, the most frequently reported of which included headache, dizziness, and nausea [186]. CX717 demonstrated clinical efficacy in treating ADHD in adults, showing a significant improvement compared to placebo. In addition, CX717 effectively reversed opioid-induced respiratory depression without compromising analgesia in human subjects [187]. The clinical relevance of CX717 in reversing cognitive deficits associated with sleep deprivation has also been investigated, but the results of these studies were not conclusive. The compound has been found to exert no significant influence on performance and alertness during night shift work in comparison to a placebo when administered in doses of 200, 400, or 1000 milligrams [188]. However, another study demonstrated that CX717 at a dose of 1000 mg counteracts the effects of sleep deprivation on attention-based tasks, particularly those involving information processing and attention maintenance [189]. A close analog of **8**, CX1739 (**9**), has demonstrated high oral bioavailability and good tolerance for single and multiple oral rising doses in young healthy volunteers in a Phase I clinical trial [186]. The compound has been subjected to a Phase II investigation regarding its ability to antagonize opioid-induced respiratory depression with preservation of opioid analgesia; however, the results have not yet been published.

ORG-26576 (**10**) has also produced inconclusive outcomes in Phase II clinical trials for the treatment of attention deficit hyperactivity disorder in adults. A 100 mg dosage administered twice daily was found to be superior to placebo in alleviating the symptoms of ADHD; however, these results were not confirmed under a higher flexible dose regimen [178]. The compound was also evaluated in a Phase II trial for its potential to treat major depressive disorder; however, the results of the trial indicated no significant efficacy in comparison with a placebo [177,190].

S47445 (**18**, CX1632)—another CX compound developed by RespireRx Pharmaceuticals—has been assessed for its capacity to address cognitive and depressive symptoms in patients with mild to moderate AD. Over a 24-week administration period, CX1632 was found to be safe and well-tolerated; however, it did not demonstrate significant advantages over placebo in terms of cognitive function, daily functioning, or depressive symptoms [179]. The compound also failed Phase II trials as an adjunctive treatment for major depressive disorder in patients with inadequate response to other antidepressant therapies [191,192].

The development of AMPAR PAM molecules by other companies has also led to their advancement into clinical trial phases. The substantial memory-enhancing effects of Servier’s cyclothiazide-related derivative, S18986 (**22**), have been demonstrated in middle-aged rodents across a variety of animal behavioral models, suggesting its potential for treating mild cognitive impairment (MCI)—a prodromal stage of AD [193]. In Phase II long-term clinical trials in MCI patients, the compound was investigated in dosage forms of 15 mg and 50 mg; however, the study was prematurely terminated due to adverse events associated with S18986 [180].

TAK-653 (**37**)—a compound developed by Takeda Pharmaceutical Company—has exhibited procognitive properties and antidepressant effects in animal models without inducing a hyperlocomotor response, which is frequently associated with the psychotomimetic side effects of ketamine in humans. A Phase II clinical trial examining compound **37** for the treatment of major depressive disorders has recently been completed, but the results have not yet been submitted. Neurocrine Biosciences has recently announced a Phase III trial regarding TAK-653 as an adjunctive treatment to antidepressants in patients with MDD. Furthermore, the pharmacodynamics of **37** in the CNS were examined in a randomized, double-blind, placebo-controlled study with 24 healthy volunteers. The findings demonstrated a psychostimulant-like pharmacodynamic profile for **37**, which was found to be generally more subtle than the clinical doses of known psychostimulants [181]. The influence of **37** on the pharmacokinetics of known CYP3A substrates (such as midazolam and ethinyl estradiol/levonorgestrel) has been evaluated in healthy adults, revealing that **37** does not lead to the induction of CYP3A and could be safely co-administrated with the mentioned drugs [194].

In human studies evaluating the ability of compounds to attenuate ketamine-induced impairment in verbal learning and memory, BIIB104 (**46**) significantly improved immediate recall and spatial working memory without notably reducing ketamine’s psychotomimetic effects [183,195]. However, the results of Phase II clinical trials in patients with schizophrenia demonstrated no significant improvements in cognitive function over placebo [184,195]. Similarly, a single-dose evaluation in patients with mild to moderate age-related sensorineural hearing loss showed no significant pharmacological effect [182].

In line with the results of preclinical studies of the compound GSK729327 (UoS12258, **47**), which exhibited a favorable pharmacological profile and therapeutic potential for enhancing cognitive abilities, the compound was subjected to a Phase I trial in patients diagnosed with schizophrenia and was proven to be safe and well-tolerated in single- and multiple-dose tests in healthy volunteers. However, further development of **47** was terminated due to its prolonged half-life and associated possibility of drug accumulation [144].

A clinical evaluation of mibampator (LY451395, **48**)—a compound developed by Eli Lilly and Company—has been conducted to assess its potential for therapeutic applications related to agitation, aggression, and cognitive deficits in patients with AD; however, no advantage of LY451395 over placebo in terms of efficacy has been reported [185,196]. It should be noted that this limited efficacy may have been due to the dosing protocol used in the study, as **48** was administered at 1/5 and 1/15 of the maximum tolerated dose due to toxicological concerns [196].

## 5. Illicit Use and Potential Abuse as ‘Smart Drugs’

Recent trends indicate a substantial rise in the use of cognitive-enhancing substances, commonly referred to as ‘smart drugs’ or ‘brain boosters,’ particularly among students. Originally developed for the treatment of neurological and psychiatric disorders—including Alzheimer’s disease and ADHD—nootropics have become a topic of increasing concern [197,198]. In recent years, there has been an escalating proliferation of websites that address this subject in a popular scientific manner, offering guidance or even providing nootropic substances. Some of the most widely used ‘smart drugs’ include amphetamine, methylphenidate, donepezil, selegiline, piracetam, modafinil, benzodiazepine inverse agonists, and natural plant preparations such as *Bacopa monnieri* and *Ginkgo biloba* [199].

Positive allosteric modulators of AMPAR based on the pyrrolidin-2-one scaffold—including piracetam, nefiracetam, and aniracetam derivatives—have gained increasing prominence as nootropics in recent years. Notably, the two piracetam analogs DM232 (unifiram) and DM235 (sunifiram)—first described in 2000 by Manetti et al. [30,31]—have attracted significant interest.

The precise mechanisms of action of sunifiram and unifiram remain incompletely understood, but some evidence suggests that their potent cognitive-enhancing properties (estimated to be up to four times greater than those of piracetam) can be attributed—at least in part—to the modulation of AMPA receptor activity. In preclinical studies, both compounds demonstrated potency in counteracting cognitive deficits; however, neither unifiram nor sunifiram has progressed to clinical trials, leaving their therapeutic potential unverified in human populations. Despite this, both have gained considerable popularity in online communities and are readily available through various online vendors, often with documentation verifying their purity. Their widespread availability has led to a significant increase in self-medication and experimentation without necessary medical supervision, with online forums serving as informal platforms for users to share their experiences, such as anecdotal clinical trials. However, this trend raises serious safety and efficacy concerns, especially given the lack of long-term clinical evaluations [33].

Despite the growing interest in nootropics, their efficacy in healthy populations remains a subject of ongoing debate. Furthermore, the long-term safety profiles of many nootropics—including unifiram and sunifiram—remain poorly understood, raising concerns regarding potential side effects such as addiction, cardiovascular risks, neurochemical imbalances, and broader implications for brain health. Ethical considerations and regulatory challenges surrounding their widespread use further complicate the discourse, necessitating more rigorous scientific inquiry and policy discussions to comprehensively assess their risks and benefits [197].

## 6. Conclusions and Future Directions

Positive allosteric modulators of AMPA and kainate receptors have emerged as promising therapeutic agents for enhancing excitatory neurotransmission, with potential applications in the treatment of neurological and psychiatric disorders such as ADHD, cognitive impairment associated with schizophrenia and AD, opiate-induced respiratory depression, and major depressive disorder. Advances in structural biology and pharmacology have led to the development of diverse PAM chemotypes, each with distinct effects on receptor desensitization and deactivation. Despite the significant progress made in terms of understanding their mechanisms of action, the clinical implementation of AMPAR PAMs remains challenging, with many having failed in later-stage trials due to safety concerns or limited efficacy.

One of the primary challenges in the development of AMPAR PAMs is the possible risk of adverse effects, particularly seizure induction and excitotoxicity. While PAMs offer more physiologically subtle modulation compared to orthosteric agonists, excessive enhancement in glutamatergic signaling can trigger neurotoxicity and convulsions. In this context, low-impact ampakines that produce more moderate enhancement of receptor functions are particularly promising for chronic therapeutic use. Such agents demonstrate excellent safety profiles, with minimal proconvulsant activity even at very high (i.e., supratherapeutic) doses. Some recent studies have also followed the strategy of designing PAMs with reduced agonist activity, thus avoiding direct receptor activation and minimizing susceptibility to seizures while maintaining therapeutic efficacy. Additionally, the design of compounds that selectively interact with specific AMPA receptor–auxiliary subunit complexes (such as TARPs and CNIHs) is considered a promising approach to enhance therapeutic precision while reducing side effects.

Another barrier to the development of clinically viable AMPA PAMs relates to pharmacokinetic limitations. Some of the early AMPA PAMs suffered from poor metabolic stability, short or prolonged half-lives, and low solubility. These issues highlight the need for the comprehensive development of more stable analogs with enhanced brain penetration ability, extended duration of action, and improved oral bioavailability. Recent advances in rational medicinal chemistry have led to the design of optimized compounds that successfully address these challenges. Through targeted structural modifications, newer analogs have demonstrated significantly better pharmacokinetic profiles, serving as promising candidates for clinical evaluation.

Kainate receptors, although less extensively studied to date, represent an emerging area of interest. The development of KAR PAMs has been hampered by the lack of subtype-selective ligands and insufficient understanding of their diverse roles in synaptic transmission and modulation. Nevertheless, recent progress involving benzothiadiazide analogs has shown promising activity at specific KAR subtypes, highlighting the feasibility and therapeutic potential of developing KAR-selective positive allosteric modulators.

In conclusion, future research should focus on optimizing AMPAR PAMs, thus providing improved selectivity, reduced side effects, and broader therapeutic windows. Targeting specific receptor–subunit complexes, auxiliary proteins, and region-specific receptor populations may enhance the precision of these modulators while minimizing side effects. Additionally, further studies on bivalent ligands, isoform-selective compounds, and novel scaffold designs could contribute to the next generation of AMPAR PAMs with enhanced clinical potential and mitigated seizure risk. At the same time, the limited availability of potent and selective KAR PAMs highlights a significant gap in the field, underscoring the need for intensified efforts to investigate their roles in CNS functioning and their possible therapeutic potential.

## Figures and Tables

**Table 1:** AMPAR potentiation effects of benzamide-type modulators derived from CX614.

Compound	EC_2X_ [μM]	A [%] (Conc. [μM] *^a^* or Dose [mg/kg] *^b^*)	EC_50_ [μM]	E_max_ (Fold Increase)
CX614 (**11**)	2.3 *^c,d^* 1.0 *^e^* 0.88 *^f^*	nd	17 *^e^* 11 *^f^*	30 *^e^* 23 *^f^*
**12** *^c,d^*	0.06	nd	nd	nd
**13** *^c,g^*	0.011	16 (3) *^a^*	nd	nd
**14** *^c,g^*	0.0007	19 (0.3) *^a^*	nd	nd
**15** *^c,h^*	0.0037	27 (3) *^b^*	nd	nd
**16** *^c,h^*	0.0031	24 (5) *^b^*	nd	nd
**17** *^c,h^*	0.46	33 (5) *^b^*	nd	nd
CX1632 (**18**)	1.5 *^e^* 4.8 *^f^*	nd	6.5 *^e^* 7.1 *^f^*	7.9 *^e^* 6.9 *^f^*

EC_2X_—concentration of drug yielding a 2-fold increase in the magnitude of the current induced by glutamate. EC_50_—modulator concentration responsible for 50% of the maximal effect. E_max_—maximal effect related to the response without the modulator. A—*^a^* in vitro slice assay: increase in the amplitude baseline evoked by the indicated concentration of the compound, recordings of the field EPSP from rat hippocampal slices, Ref. [67]; *^b^* in vivo electrophysiology assay: increase in the amplitude baseline evoked by the indicated dose of the compound (i.p.), EPSP recordings in the dentate gyrus of anesthetized rats, Ref. [69]. *^c^* Effects on glutamate-induced (500 µM) currents in cultured rat hippocampal neurons, whole-cell patch clamp electrophysiology assay; *^d^* Ref. [67]; *^e^* effects on AMPA-induced (10 µM) currents in *Xenopus laevis* oocytes injected with rat cortex mRNA, voltage clamp assay, ref. [70]; *^f^* effects on AMPA-induced (10 µM) currents in *Xenopus laevis* oocytes injected with human cortex mRNA, voltage clamp assay, ref. [70]; *^g^* Ref. [68]; *^h^* Ref. [69]; nd—not determined.

**Table 2:** AMPAR potentiation effects of benzothiadiazine-type modulators.

Compound	EC_2X_ *^a^* ^or *b*^ [μM]	EC_50_ [μM]	E_max_ [%]
Cyclothiazide (**20**)	1.6 *^a,c^*	7.1 *^c^*	844 *^c^*
IDRA-21 (**21**)	134 *^a,c^*	nd	>700 *^c^*
S18986 (**22**)	25 *^a,c^*	130 *^c^*	1263 *^c^*
BPAM97 (**23**)	3.2 *^a,c^*	33 *^c^*	4066 *^c^*
BPAM121 (**24**)	19 *^b,d^* 6.7 *^a,d^*	nd	nd
BPAM344 (**25**)	0.27 *^b,e^*	0.90 *^e^*	1500 *^e^*
BPAM521 (**26**)	0.5 *^b,f^*	nd	620 *^f^*
BPAM549 (**30**)	2.2 *^b,f^*	nd	850 *^f^*
BPAM395 (**31**)	0.24 *^b,g^*	4.7 *^g^*	nd
BPAM279 (**32**)	34 *^b,g^*	40 *^g^*	nd

*^a^* concentration of drug yielding a 2-fold increase in (*S*)-AMPA-induced (10 μM) current amplitude in *Xenopus* oocytes injected with mRNA from the rat cortex, voltage clamp method; *^b^* concentration of drug yielding a 2-fold increase in the fluorescence induced by (*S*)-AMPA (300 μM) in primary cultures of neurons from the rat cortex; *^c^* voltage clamp method, Ref. [81]; *^d^* Ref. [82]; *^e^* fluorescence assay, Ref. [83]; *^f^* fluorescence assay, ref. [84]; *^g^* fluorescence assay, Ref. [85]; nd—not determined.

**Table 4:** Comparison of potentiation effects of IDRA-21 and -**33** measured via electrophysiological recordings at native KARs as well as recombinant homomeric and heteromeric KARs and AMPARs (adapted from Ref. [97]).

Receptor		EC_50_ [μM]		E_max_ [%]	
		IDRA-21 (21)	33	IDRA-21 (21)	33
Native kainate receptors	KA *^a^*	133	8	99	177
	KA + GYKI *^b^*	568	20	375	500
Recombinant homomeric and heteromeric iGluRs	GluA1 *^c^*	585	70	300	1600
	GluA2 *^c^*	532	47	105	529
	GluK1 *^d^*	590	105	180	275
	GluK1/4 *^d^*	690	550	290	660
	GluK1/5 *^d^*	340	338	283	710
	GluK2 *^e^*	688	190	400	800
	GluK2/4 *^e^*	238	206	92	657
	GluK2/5 *^e^*	714	195	293	448

*^a^* effects on the KA-induced (100 μM) current in cerebellar granule cells; *^b^* effects on the KA-induced (100 μM) current in cerebellar granule cells in the presence of 100 μM GYKI 53655; *^c^* effects on the glutamate-induced (100 μM) current in the HEK293 cell line expressing recombinant homomeric GluA1 and GluA2 receptors; *^d^* effects on the glutamate-induced (100 μM) current in the HEK293 cell line expressing the GluK1 subunit (homomeric GluK1 receptors or GluK1/4 and GluK1/5 heteromeric receptors); *^e^* effects on the glutamate-induced (100 μM) current in the HEK293 cell line expressing the GluK2 subunit (homomeric GluK2 receptors or GluK1/4 and GluK1/5 heteromeric receptors).

**Table 5:** AMPAR potentiation effects of compounds TAK-137 and TAK-653 (adapted from Ref. [101]).

Compound	Intracellular Ca^2+^ Influx Assay		Electrophysiological Recordings	
	Potentiation EC_50_ (μM) *^a^*	Agonistic Effect (%) *^c^*	Potentiation EC_50_ (μM) *^b^*	Agonistic Effect (%) *^c^*
TAK-137 (**36**)	0.42	7.6	1.4	6.4
TAK-653 (**37**)	0.93	4.8	4.4	1.7

*^a^* effects on AMPA-induced (5 μM) fluorescence in primary cultures of rat neurons (intracellular Ca^2+^ influx assay); *^b^* effects on the AMPA-induced (1 μM) current in rat primary hippocampal neurons, whole-cell patch clamp recordings; *^c^* agonistic effect of the compound (30 μM) in the absence of AMPA, expressed as a percentage of the maximal response induced by 5 μM AMPA (Ca^2+^ assay) or 100 μM AMPA (patch clamp method).

**Table 8:** Potentiation effects of selected sulfonamide analogs at recombinant homomeric AMPA receptors.

Compound	EC_50_ [µM] (E_max_ [%])						
	GluA1_i_	GluA1_o_	GluA2_i_	GluA2_o_	GluA3_i_	GluA4_i_	GluA4_o_
LY451395 (**48**) *^a^*	0.5	2.2	nd	nd	nd	0.40	1.9
PIMSD (**49a**) *^b^*	1.52 (1335)	nd	0.73 (790)	0.64 (804)	1.90 (2217)	0.87 (383)	nd
CMPDA (**50**) *^c^*	nd	nd	0.045	0.063	nd	nd	nd
CMPDB (**51**) *^c^*	nd	nd	0.12	0.47	nd	nd	nd

*^a^* effects on glutamate-evoked (10 mM) current in HEK293 cells expressing GluA1 and GluA4 receptors (in flip and flop variants), voltage clamp method, Ref. [7]; *^b^* effects on glutamate-evoked (10 µM) response in *Xenopus laevis* oocytes expressing recombinant rat AMPARs, voltage clamp method, Ref. [134]; *^c^* effects on glutamate-evoked (100 µM) current in HEK293 cells expressing homomeric GluA2_i_ and GluA2_o_ receptors, Ca^2+^ influx assay, Ref. [135]; nd—not determined.

**Table 9:** Potentiation effects of trifluoromethylpyrazole analogs at native and recombinant AMPARs, measured via intracellular calcium assay.

Compound	Native AMPAR	GluA1_i_	GluA2_i_
	EC_50_ [µM]	EC_50_ [µM]	EC_50_ [µM] (E_max_ [%])
**55** *^a^*	nd	0.25	nd
**56** *^a^*	nd	0.79	nd
JAMI1001A (**57**) *^a^*	nd	0.39	nd
**58** *^a^*	nd	0.50	nd
**59** *^a^*	nd	0.50	nd
HBT1 (**60**)	1.3 *^b^*	4.6 *^c^*	nd
**61** *^d,e^*	nd	nd	25.1 (94)
MDI-222 (**62**) *^d,f^*	nd	22.4 (30)	5.0 (123)

*^a^* effects on the glutamate-induced (50 μM) response in HEK293 cells, Ref. [139]; *^b^* effects on the AMPA-induced (5 µM) response in primary neurons, Ref. [132]; *^c^* effects on the glutamate-induced (3 mM) response in CHO cells, Ref. [132]; *^d^* effects on the glutamate-induced (100 μM) response in HEK293 cells; *^e^* Ref. [143]; *^f^* Ref. [144]; nd—not determined.

## Data Availability

Not applicable.
